# Supplementary material for: Nrf1 Is Endowed with a Dominant Tumor-Repressing Effect onto the Wnt/β-Catenin-Dependent and Wnt/β-Catenin-Independent Signaling Networks in the Human Liver Cancer
Source: Oxid Med Cell Longev. 2020 Mar 23;2020:5138539. doi: 10.1155/2020/5138539 (PMC7125503; doi:10.1155/2020/5138539)
Supplement: Supplementary 1 — Figure S1: expression of Nrf1/TCF11 and NQO1 in three pairs of shNrf1- and shNC-expressing cell lines that were treated with 10 μmol/L of MG132 or vehicle control (DMSO) for 4 h. Figure S2: distinct expression levels of multiple Nrf1 isoforms. Figure S3: significant increments in the migration and invasion of Nrf1-silenced hepatoma cells. Figure S4: obvious changes in shNrf1-expressing cell viability, colony formation, cell cycle, and its apoptotic rate. Figure S5: comparison of differential expression genes in distinct genotypic cell lines. Figure S6: context-dependent expression of Wnt/β-catenin signaling responsive genes on distinct extents of Nrf1 deficiency in human hepatomas with different differentiation. Figure S7: scatter plot of the enriched KEGG pathways. Figure S8: significant differences in the expression of genes involved in cancer related pathway, focal adhesion, and ECM-receptor interaction. Figure S9: aberrant activation of the PI3K-PDK1-AKT signaling in Nrf1-deficient hepatoma cells. [file 5138539.f1.pdf]

Nrf1 is endowed with a dominant tumor-repressing effect onto the Wnt/β-Catenin - dependent and -independent signaling networks in the human liver cancer

Jiayu Chen[1,2,§], Meng Wang[1,§], Yuancai Xiang[1,3,§], Xufang Ru[1,4], Yonggang Ren[1,5], Xiping Liu[2], Lu Qiu[1,6], and Yiguo Zhang[1*]

[1]The Laboratory of Cell Biochemistry and Topogenetic Regulation, College of Bioengineering and Faculty of Sciences, Chongqing University, No. 174 Shazheng Street, Shapingba District, Chongqing 400044, China. [2]Department of Biochemistry and Molecular Biology, Zunyi Medical University, No. 6 Xuefu-Xi Road, Xinpu New District, Zunyi 563000, Guizhou, China. [3]Department of Biochemistry and Molecular Biology, School of Basic Medical Sciences, Southwest Medical University, No. 1 at the First Section of Xianglin Road, Longmatan District, Luzhou 646000, Sichuan, China. [4]Department of Neurosurgery, Southwest Hospital, Army (Third Military) Medical University, No. 29 Gaotanyan Street, Shapingba District, Chongqing 400038, China. [5]Department of Biochemistry, North Sichuan Medical College, No. 55 Dongshun Road, Gaoping District, Nanchong 637000, Sichuan, China. [6]School of Life Sciences, Zhengzhou University, No. 100 Kexue Avenue, Zhengzhou 450001, Henan, China.

[§]Contributed equally to this work.

*Correspondence should be addressed to Yiguo Zhang (Email: yiguozhang@cqu.edu.cn, or eaglezhang64@gmail.com)

Figure S1

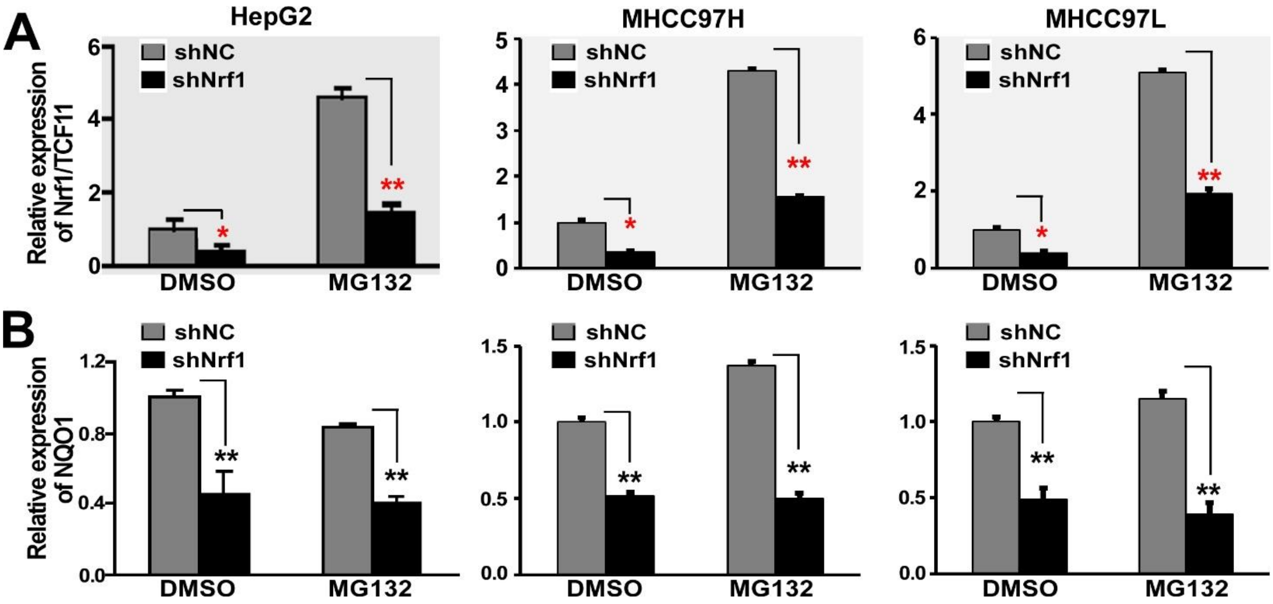

Figure S1. Expression of Nrf1/TCF11 and NQO1 in three pairs of shNrf1- and shNC-expressing cell lines that were treated with 10 µmol/L of MG132 or vehicle control (DMSO) for 4 h. The intensity of those immunoblots (as shown in Figure 1C) was quantified by the Quantity One 4.5.2 software and are shown graphically. The data are shown as mean ± SD (n = 3), with significant decreases of shNrf1 (* $p < 0.05$; ** $p < 0.01$), when compared to the shNC values.

Figure S2

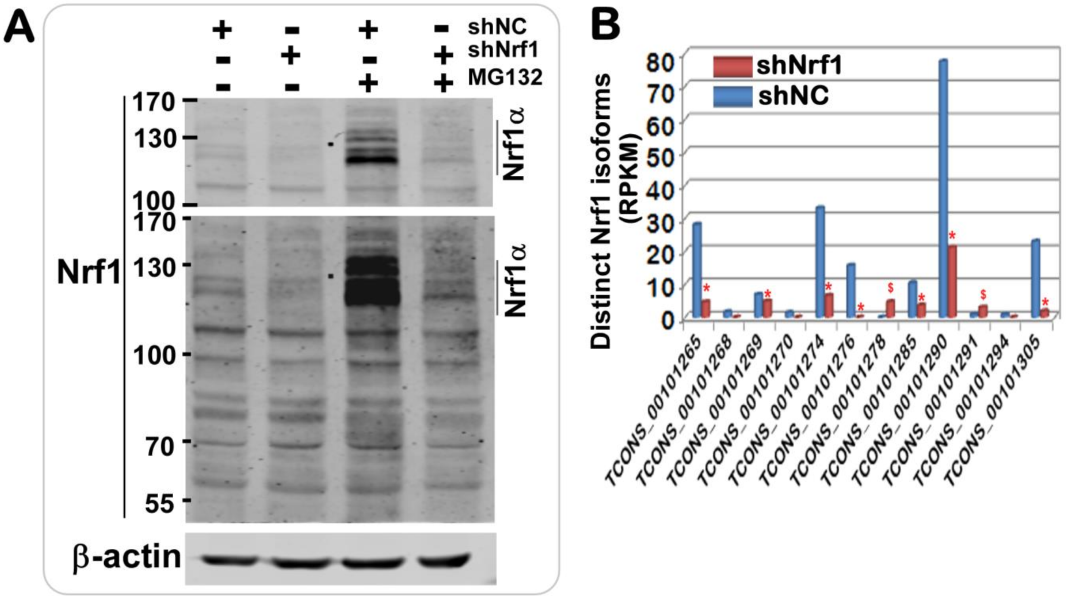

C Distinct isoforms of Nrf1 detected in shNrf1- and shNC-expressing HepG2 cells

Test-ID (Nrf1 isoforms)	shNC (RPKM)	%	shNrf1(RPKM)	%	shNrf1-shNC
TCONS_00101265	28.1053	14.06	4.6589	2.33	**-23.446**
TCONS_00101268	1.6880	0.84	0.0000	0.00	**-1.688**
TCONS_00101269	6.9538	3.48	4.9050	2.45	**-2.049**
TCONS_00101270	1.4663	0.73	0.0000	0.00	**-1.466**
TCONS_00101274	33.1425	16.58	6.6064	3.31	**-26.536**
TCONS_00101276	15.7253	7.87	0.0000	0.00	**-15.725**
TCONS_00101278	0.0000	0.00	4.7126	2.36	**4.713**
TCONS_00101285	10.4507	5.23	3.7043	1.85	**-6.746**
TCONS_00101290	77.5503	38.81	21.1819	10.60	**-56.368**
TCONS_00101291	0.8901	0.45	3.0687	1.54	**2.179**
TCONS_00101294	0.8251	0.41	0.0000	0.00	**-0.825**
TCONS_00101305	23.0460	11.53	1.8938	0.95	**-21.152**
**Total 12 isoforms**	**199.8434**	**99.99**	**50.7316**	**25.39**	**-149.112**

Figure S2. Distinct expression levels of multiple Nrf1 isoforms. (**A**) The HepG2 cells, that had been allowed for stably expression of shNrf1 or shNC packaged in a lentivirus-transduced system, were treated with 10 μmol/L of MG132 (+) or a vehicle DMSO (–) for 24 h before being subjected to evaluation of distinct Nrf1 isoforms by Western blotting with a specific antibody against Nrf1 (made in our own laboratory). (**B, C**) Both the histogram (*B*) and table (*C*) of the sequencing reads that distribute to a reference in the concrete and complete genome-wide level of human *Nrf1* gene with distinct transcripts that were compared between two samples of shNrf1- and shNC-expressing HepG2 cell lines. Knockdown of Nrf1 by shNrf1 caused significant decreases (* $p < 0.05$; ** $p < 0.01$) or significant increases ($, $p < 0.05$) relative to the counterpart values measured from shNC cells.

Figure S3

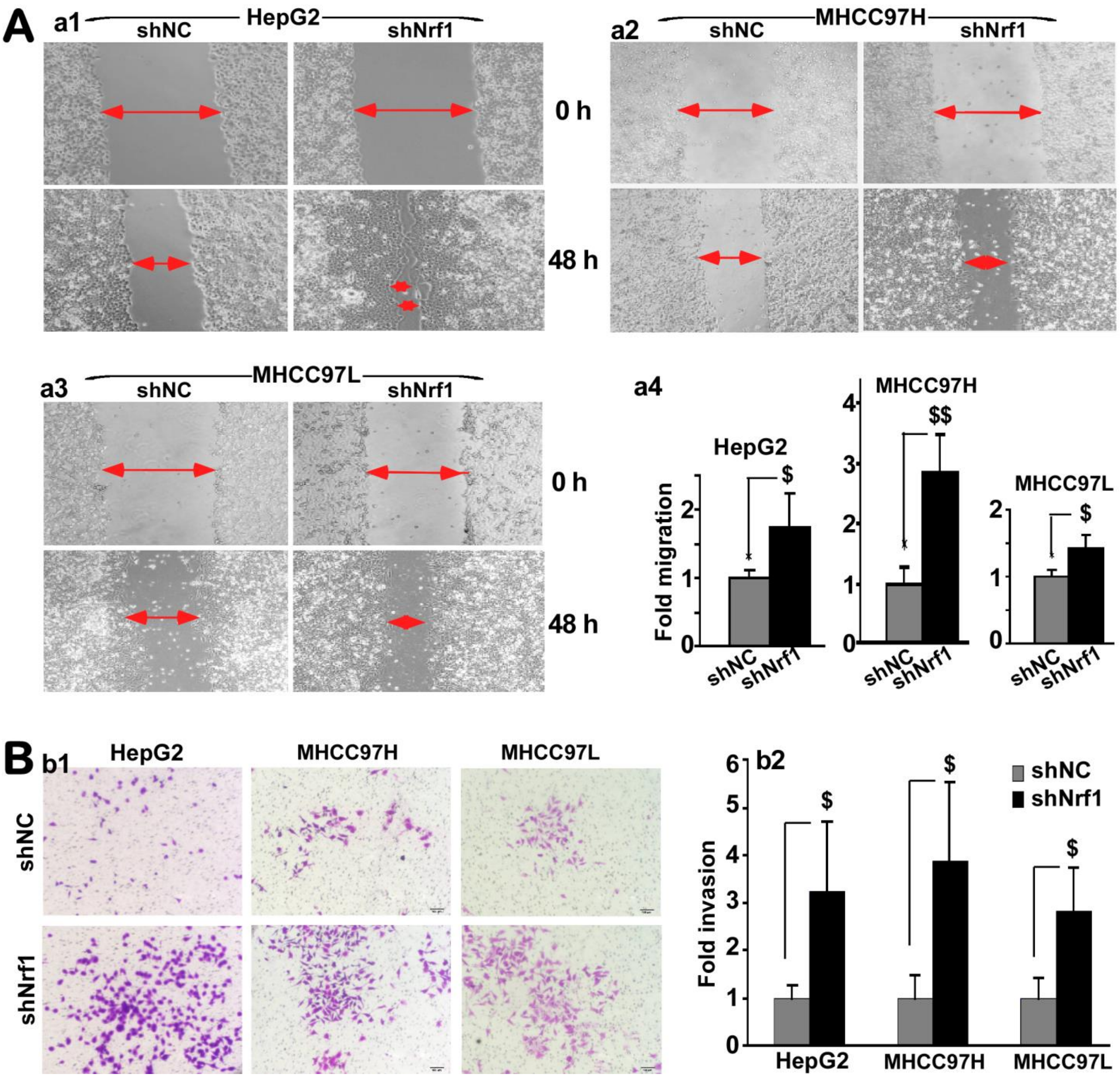

Figure S3. Significant increments in the migration and invasion of Nrf1-silenced hepatoma cells. (**A**) The migration to close the scratched edges (100 ×) of hepatoma cell lines HepG2 (a1), MHCC97H (a2) and MHCC97L (a3), which had been infected by the lentivirus expressing shNrf1 or shNC, was quantified and shown graphically (a4). (**B**) The Matrigel invasion assay of three distinct hepatoma cell lines expressing shNrf1 or shNC was conducted, and the photographed results (100 ×, b1) were quantified as shown graphically (b2). The error bars (a4 and b2) represent the mean ± SD of three independent experiments in triplicates (n = 3 × 3). Significant increases ($, p < 0.05; $$, p < 0.01) were determined by one-way ANOVA analysis, compared to shNC controls.

Figure S4

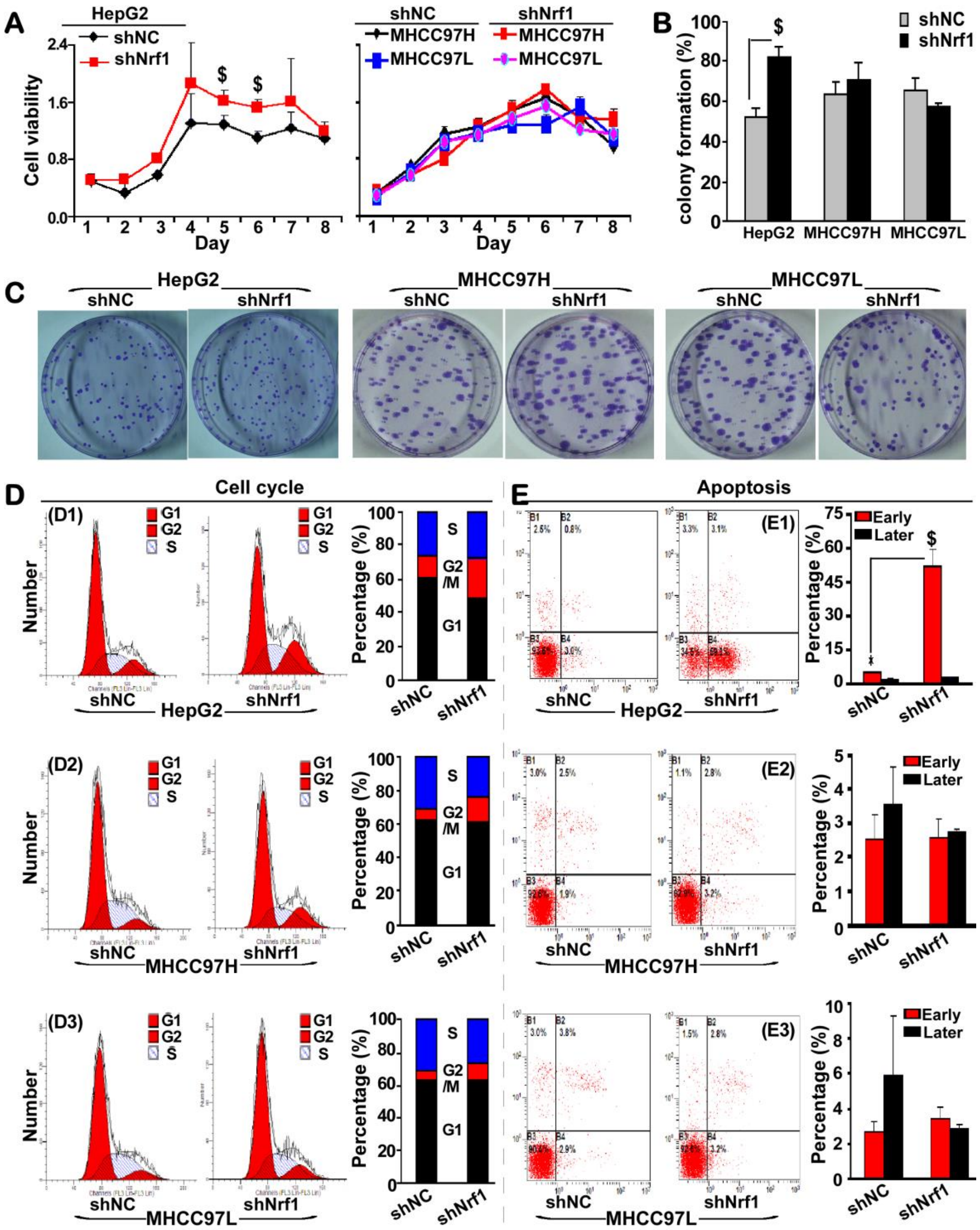

Figure S4. Obvious changes in shNrf1-expressing cell viability, colony formation, cell cycle, and its apoptotic rate. (**A**) The growth curves of shNrf1- and shNC-expressing cell lines was shown after the MTT analysis of their viabilities. (**B**, **C**) The *in vitro* colony formation of shNC and shNrf1 cell lines. The resulting cell colonies on the plates were stained with 1% crystal violet reagent before being counted. The data were calculated as a fold change (mean ± SD, n = 3; $, $p < 0.05$) of the shNrf1-derived clone formation, relative to the shNC controls. (**D**) Changes in the above hepatoma cell cycles were analyzed by fluorescent-activated cell sorting (FACS), and are shown in the percent columns. (**E**) The apoptosis was measured by FACS with propidium iodide (PI)-stained or Annexin V-stained cell lines as indicated. The column data analysis (n = 3) of their early apoptotic (Annexin V^+, PI^-) and late apoptotic (and/or necrotic) cells (Annexin $V2^+$, PI^+) in each group. Significant increases ($, $p < 0.05$) in shNrf1 cells were compared to the shNC controls.

Figure S5

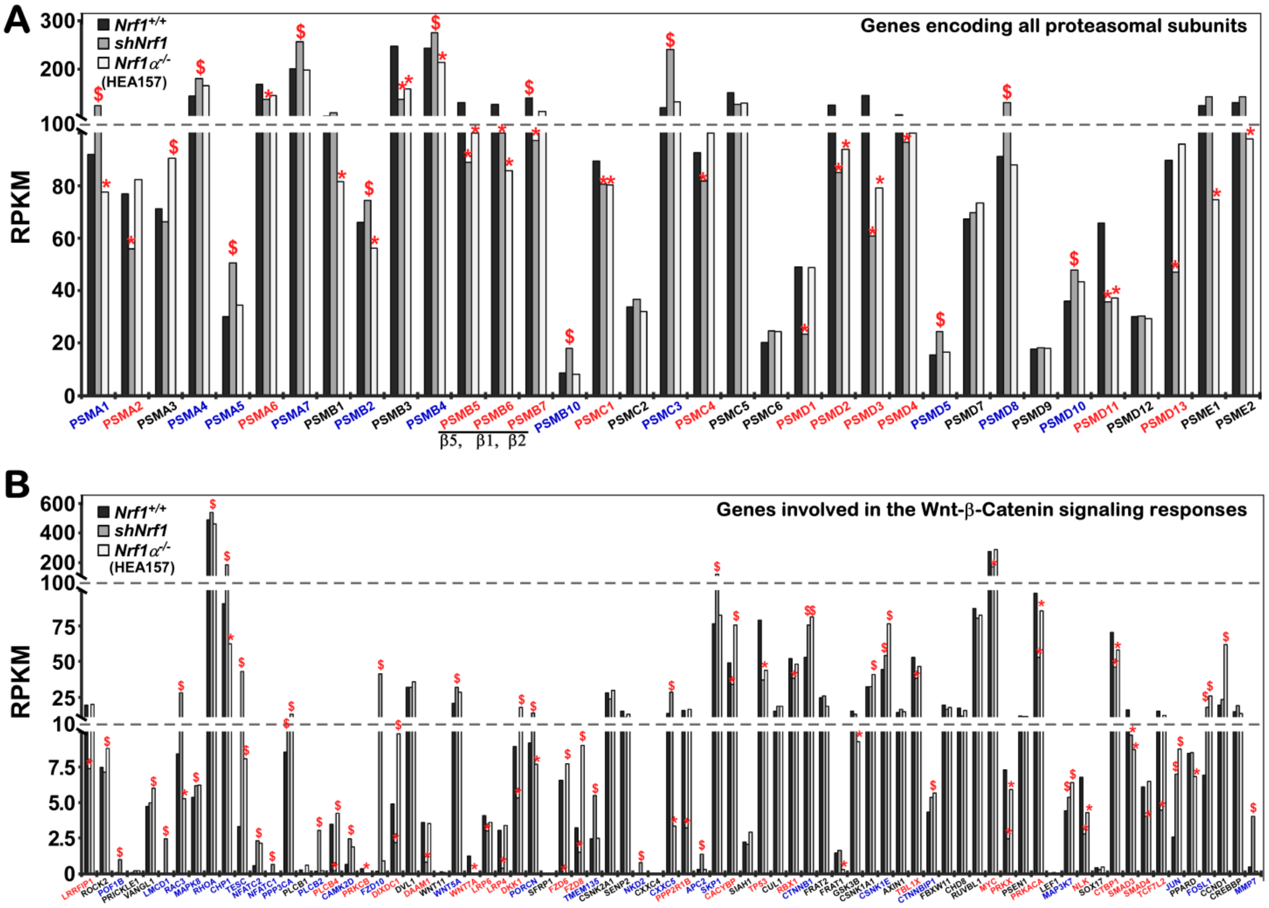

Figure S5. Comparison of differential expression genes in distinct genotypic cell lines. (A) Differential expression levels of distinct proteasomal subunits and **(B)** other genes involved in the Wnt/β-catenin signaling responsive cascades, were determined by transcriptomic sequencing of *Nrf1*$^{+/+}$, *shNrf1* and *Nrf1α*$^{-/-}$ (i.e., HEA157) cells. The data were subjected to statistic analysis. Significant increases ($, $p < 0.05$; $$, $p < 0.01$) and significant decreases (* $p < 0.05$; ** $p < 0.01$) were caused by deficiency of Nrf1, when compared to the counterpart values measured from *Nrf1*$^{+/+}$ cells.

Figure S6

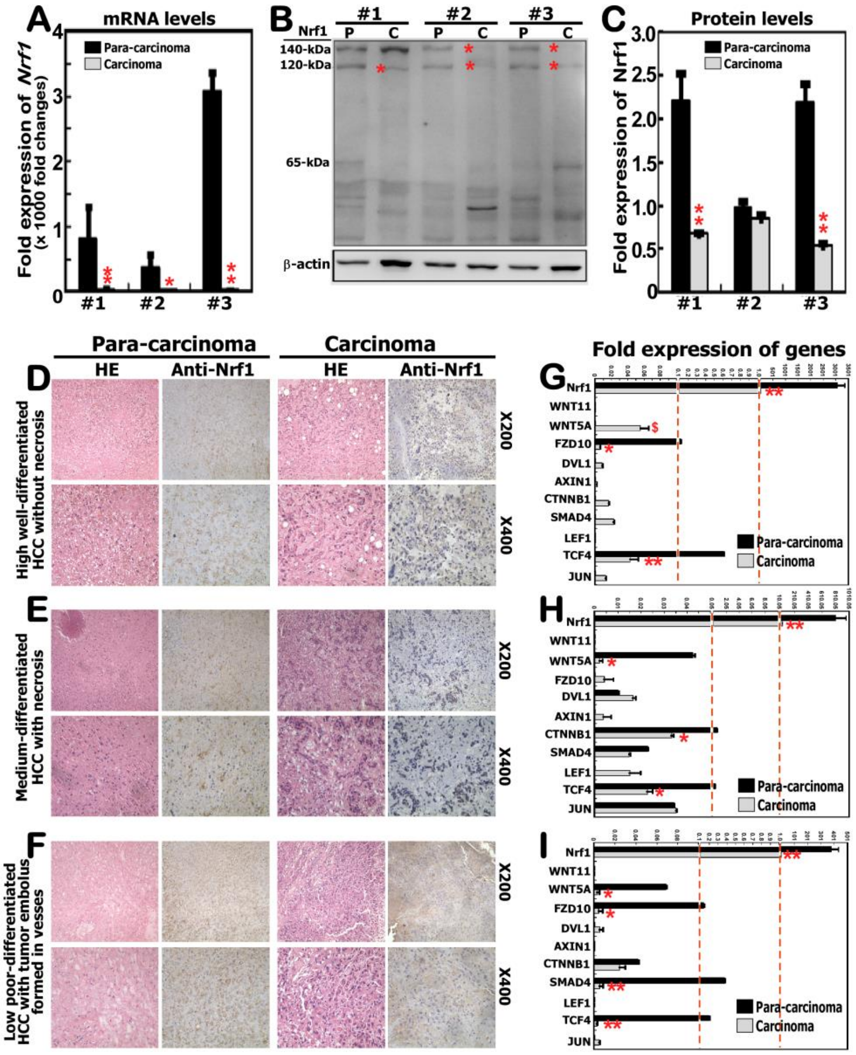

Figure S6. Context-dependent expression of Wnt/β-catenin signaling responsive genes on distinct extents of Nrf1 deficiency in human hepatomas with different differentiation. Distinct expression profiles of Nrf1 at its mRNA levels (**A**) and protein levels (**B, C**) were determined respectively by real-time qPCR and Western blotting of three pairs of human hepatocellular carcinoma (HCC) and adjacent para-carcinoma tissues that had been pathologically diagnosed at distinct stages of cancer development and progression. (**D** to **F**) Their pathohistological photographs and (**G** to **I**) mRNA expression profiles of those genes implicated in the putative Wnt/β-catenin signaling pathway in distinct HCC and adjacent para-carcinoma tissues were obtained as described in the detail of 'Materials and Methods'. Of note, (**D**, **E**) Show a high well-differentiated HCC without necrosis. (**F, G**) Represent a medium-differentiated HCC with necrosis. (H, I) Display a low poor-differentiated HCC with tumor embolus formed in vessels. The data obtained from real-time qPCR are show as fold changes (mean ± SEM, n = 3 × 3). Then statistic analysis reveals that significant increases ($, $p < 0.05$) and significant decreases (* $p < 0.05$; ** $p < 0.01$) were determined by comparison of these two grouped data measured from the core cancer and para-carcinoma tissues as indicated.

Figure S7

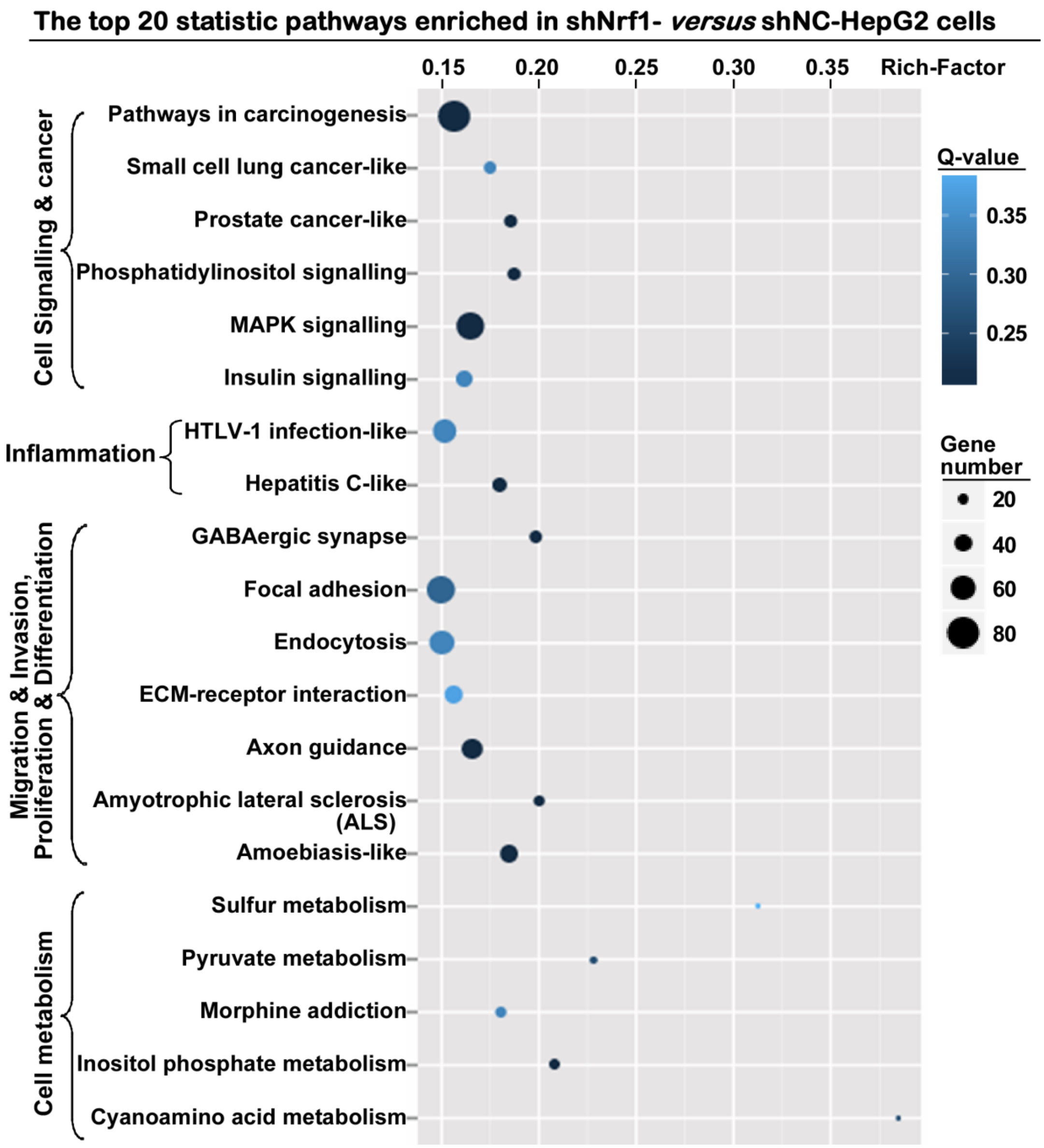

Figure S7. Scatter plot of the enriched KEGG pathways. They were enriched statistically by comparison of shNrf1- and shNC-expressing hepatoma cells. Of note, the Q-values and gene numbers were also illustrated.

Figure S8

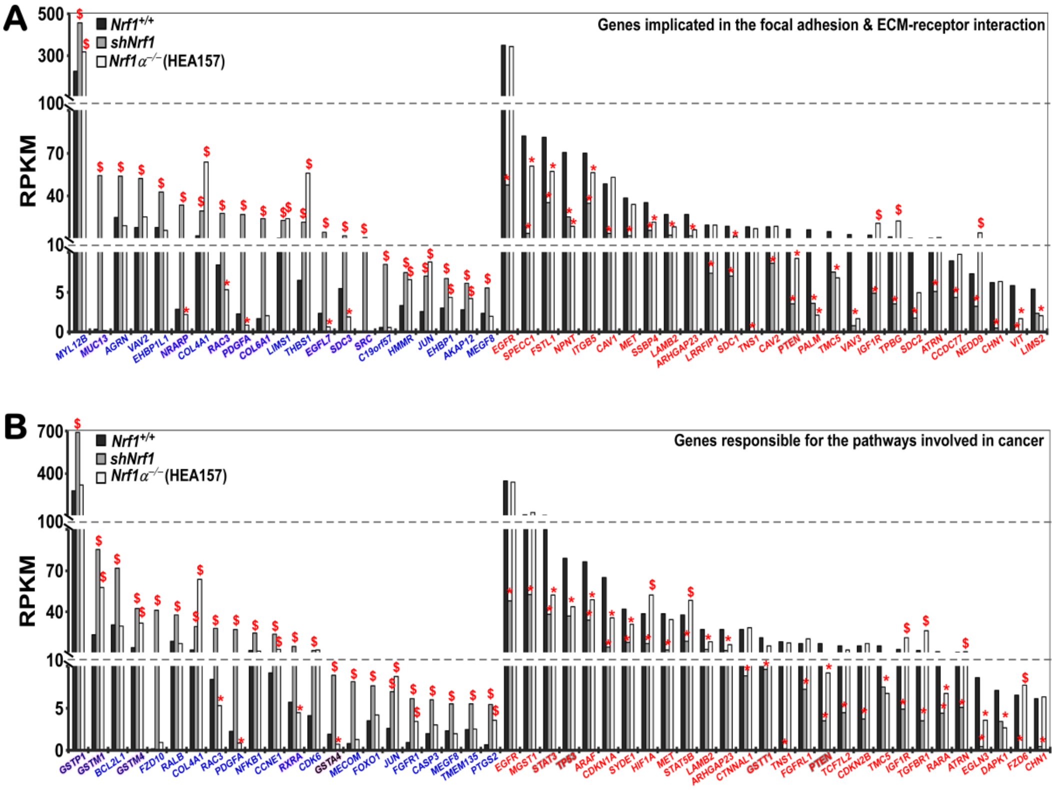

Figure S8. Significant differences in the expression of genes involved in cancer related pathway, focal adhesion and ECM-receptor interaction. The transcriptomic sequencing of *Nrf1*$^{+/+}$, *shNrf1* and *Nrf1*$\alpha^{-/-}$ (i.e., HEA157) cells reveals that amongst DEGs, those critical genes responsible for remodeling cell adhesion together with the ECM-receptor interaction (*A*) and other genes involved in cancer development through potential signaling pathways (B). The data were also subjected to statistic analysis. Significant increases ($, $p < 0.05$; $$, $p < 0.01$) and significant decreases (* $p < 0.05$; ** $p < 0.01$) were caused by deficiency of Nrf1, when compared with the counterpart values of *Nrf1*$^{+/+}$ cells.

Figure S9

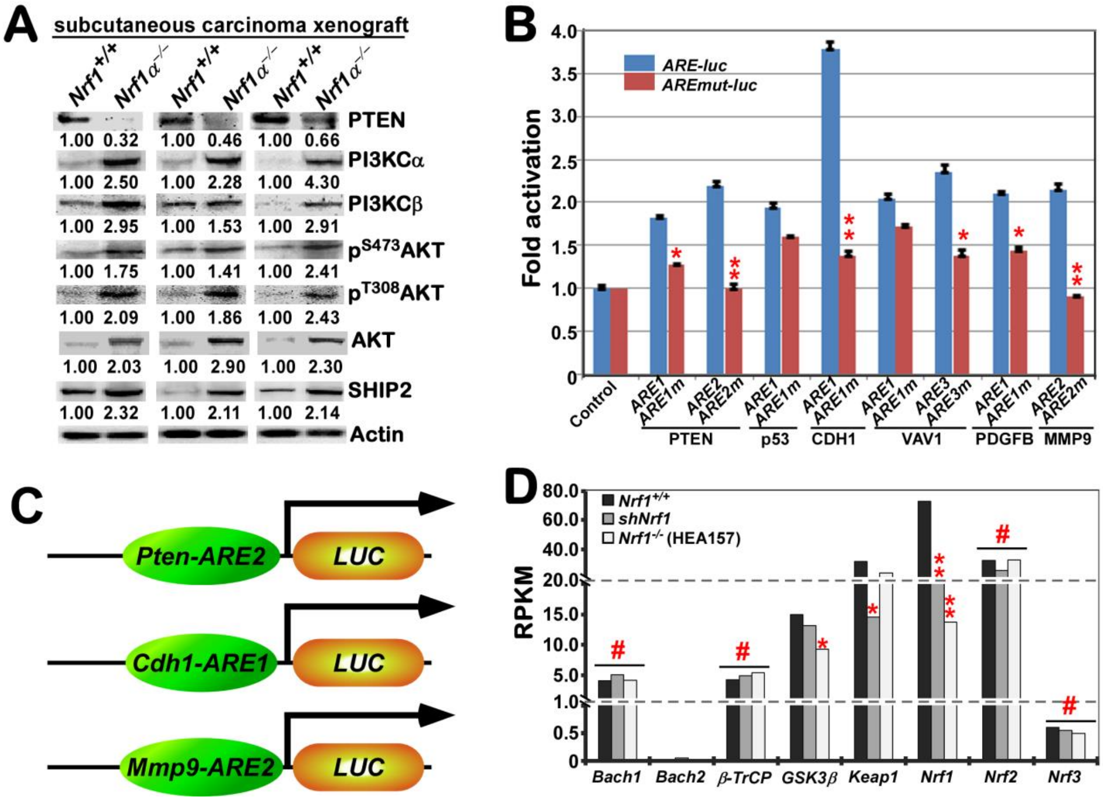

Figure S9. Aberrant activation of the PI3K-PDK1-AKT signaling in Nrf1-deficient hepatoma cells. (**A**) Several protein abundances of PTEN, PI3KCα, PI3KCβ, p^{S473}AKT, p^{T308}AKT, AKT and SHIP2 were by Western blotting of *Nrf1*$^{+/+}$- and *Nrf1α*$^{-/-}$-derived tumors that had been obtained from the subcutaneous xenograft model mice. (**B**) Eight ARE-driven luciferase reporters and their respective mutants were created by cloning of both the indicated ARE enhancer and its mutant sequences from distinct gene promoter regions of *PTEN, p53*, *CDH1*, *VAV1*, *PDGFB*, and *MMP9*. Each of these ARE-driven reporter genes or mutants, along with the pRL-TK reporter, and another expression construct for Nrf1 or an empty pcDNA3, were co-transfected into *Nrf1*$^{+/+}$ HepG2 cells and allowed for a 24-h recovery from transfection, before the luciferase activity was measured. The data are shown as mean ± SEM (n = 3 × 3). Significant decreases (* $p < 0.05$; ** $p < 0.01$) of indicated *ARE-luc* transcriptional activity were caused by each of the corresponding mutants. (*C*) Schematic representation of the *PTEN-luc*, *CDH1-luc*, *MMP9-luc* reporters. Distinct gene promoter regions of *PTEN, CDH1* and *MMP9*, as well as their core ARE sequences, were cloned into the PGL3-Promoter vector (i.e., *PGL3-Pro*) (**D**) The expression levels of *Nrf1*, *Nrf2* and their homologous genes were determined by transcriptomic sequencing of *Nrf1*$^{+/+}$*-, shNrf1* and *Nrf1α*$^{-/-}$-derived hepatoma cells. Deficiency of Nrf1 leads to significant decreases (* $p < 0.05$; ** $p < 0.01$) as indicated by comparison with the RPKM value measured from *Nrf1*$^{+/+}$ cells.

Figure S10

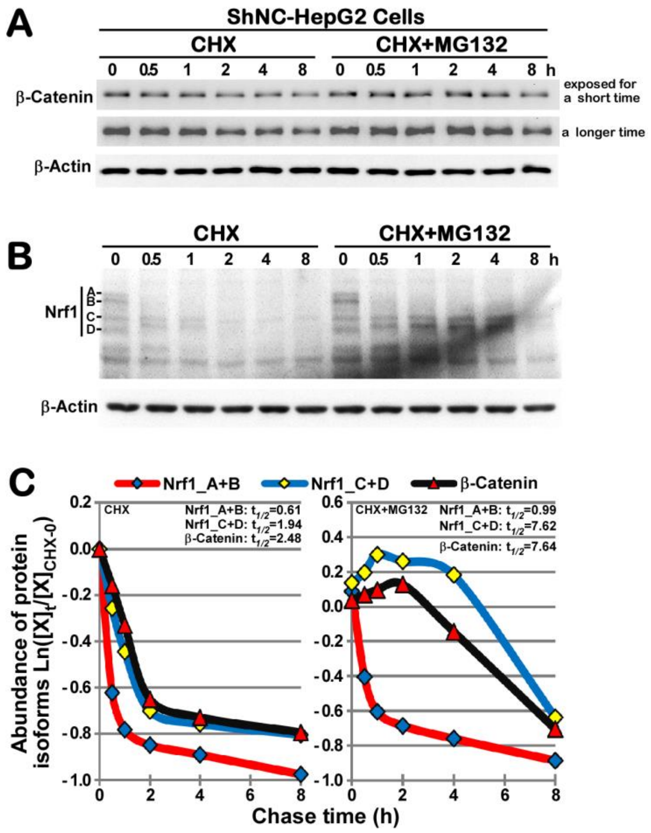

Figure S10. The stability of endogenous β-catenin and Nrf1 proteins. Experimental cells were treated with CHX (100 μg/ml) alone or in combination with MG132 (10 μmol/L) for distinct indicated lengths of time before being harvested. Subsequently, the protein abundances of β-catenin (**A**) and Nrf1 (**B**) were determined by Western blotting with their respective antibodies. The intensity of their immunoblots were quantified by the ImageJ software, and the data are shown graphically (**C**). The $[X]_t/[X]_{CHX\text{-}0h}$ represents a relative amount of indicated proteins at the indicated times 't' that had been normalized to the cells only treated with CHX for 0 h. Notably, the stability of β-catenin and Nrf1 (with distinct isoforms) was estimated by their half-lives ($t_{1/2}$) after treatment of cells with CHX alone or plus MG132.
