# Supplementary material for: Nrf1 Is Endowed with a Dominant Tumor-Repressing Effect onto the Wnt/β-Catenin-Dependent and Wnt/β-Catenin-Independent Signaling Networks in the Human Liver Cancer
Source: Oxid Med Cell Longev. 2020 Mar 23;2020:5138539. doi: 10.1155/2020/5138539 (PMC7125503; doi:10.1155/2020/5138539)
Supplement: Supplementary 2 — Table S1: the key resources used in this work. Table S2: the sequencing data of genes encoding proteasomal subunits. Table S3: the sequencing data of genes involved in Wnt/β-catenin signaling pathway. Table S4: the promoters, the enhancer ARE/AP1-binding sequences, and the corresponding mutation sequences of the representative genes of Wnt/β-catenin signaling components. Table S5: the sequencing data of genes involved in the interactive network of Nrf1 interactors, migration and invasion pathways, carcinoma related pathways, signal transduction pathways, and metabolism pathways. Table S6: the sequencing data of DEGs whose RPKM values are greater than 3 in at least one cell line (shNC- or shNrf1-HepG2). Table S7: the sequencing data of genes implicated in the focal adhesion and ECM-receptor interaction. Table S8: the sequencing data of genes responsible for the pathways involved in cancer. Table S9: the promoters, the enhancer ARE-binding sequences, and the corresponding mutation sequences of PTEN, p53, CDH1, VAV1, PDGFB, and MMP9. [file 5138539.f2.zip › Table S1_The key resources used in this work.docx]

| **Table S1.** The key resources used in this work | | |
| --- | --- | --- |
| **Reagent or Resource** | **Source** | **Identifier** |
| **Antibodies** |  |  |
| AKT | Abcam | ab32505 |
| C-Myc | Santa Cruz Biotechnology | sc-70469 |
| Cyclin D1 | Epitomics | C08053M |
| E-cadherin (CDH1) | Signalway Antibody | 48907 |
| Flag | Beyotime Biotechnology | AF519 |
| GAPDH | Signalway Antibody | 44301 |
| Lamin B1 | Signalway Antibody | 40413 |
| MMP7 | Signalway Antibody | 32086 |
| MMP9 | Signalway Antibody | 29091 |
| NQO1 | Signalway Antibody | 41254 |
| Nrf1 | Zhang’s | ([1](#_ENREF_1)) |
| PI3KCα | Bioss | bs-2067R |
| PI3KCβ | bs-1233R | bs-10657R |
| p^S473^AKT | Cell Signaling Technology | 4060 |
| p-Ser33 | Signalway Antibody | 12806 |
| p-Ser37 | Signalway Antibody | 11219 |
| p^T380^AKT | Cell Signaling Technology | 13038 |
| PTEN | Abcam | ab32199 |
| SHIP2 | Bioss | bs-1233R |
| TCF4 | Signalway Antibody | 32177 |
| TCF11/hNrf1 | Cell Signaling Technology | D5B10 |
| Tubulin | Signalway Antibody | 48885 |
| Ub | Cell Signaling Technology | 3933 |
| V5 | Ivitrogen | R960-25 |
| Vimentin | Signalway Antibody | 41532 |
| β-actin | ZSGB-BIO | TA-09 |
| β-catenin | Epitomics | K67109M |
| **Chemicals** |  |  |
| CHX (cycloheximide) | Solarbio | C8030 |
| Crystal violet | Sangon | A100528 |
| MG132 | Sigma Aldrich | M7449 |
| Penicillin-Streptomycin | Invitrogen | 15140122 |
| PMSF | Sangon | A100754 |
| Polybrene | Sigma Aldrich | TR-1003 |
| Protease inhibitors | Roche | 3271382-1 |
| Puromycin | Sigma Aldrich | P8833 |
| RIPA | Beyotime | P0013C |
| **Oligonucleotides for small hairpin (sh) or small interference (si) RNA** | | |
| shNC FW | GENECHEM | CCGGTTCTCCGAACGTGTCACGTCTCGAGACGTGACACGTTCGGAGAATTTTTG |
| shNC REV | GENECHEM | GATCCAAAAATTCTCCGAACGTGTCACGTCTCGAGACGTGACACGTTCGGAGAA |
| shNrf1(468) FW | GENECHEM | CCGGCCACAACCTGAGGAATACCTTCTCGAGAAGGTATTCCTCAGGTTGTGGTTTTTG |
| shNrf1(468) REV | GENECHEM | GATCCAAAAACCACAACCTGAGGAATACCTTCTCGAGAAGGTATTCCTCAGGTTGTGG |
| shNrf1(469) FW | GENECHEM | CCGGCGGTGAAGATTTGGAGGATTTCTCGAGAAATCCTCCAAATCTTCACCGTTTTTG |
| shNrf1(469) REV | GENECHEM | GATCCAAAAACGGTGAAGATTTGGAGGATTTCTCGAGAAATCCTCCAAATCTTCACCG |
| shNrf1(470) FW | GENECHEM | CCGGGGGATTCGGTGAAGATTTGTTCAAGAGACAAATCTTCACCGAATCCCTTTTTG |
| shNrf1(470) REV | GENECHEM | GATCCAAAAAGGGATTCGGTGAAGATTTGTCTCTTGAACAAATCTTCACCGAATCCC |
| siNC FW | GenePharma | UUCUCCGAACGUGUCACG |
| siNC REV | GenePharma | ACGUGACACGUUCGGAGA |
| siNrf1 FW | GenePharma | CCCAGCAAUUCUACCAGCCUCAACU |
| siNrf1 REV | GenePharma | AGUUGAGGCUGGUAGAAUUGCUGGG |
| **Oligonucleotides for qPCR** |  |  |
| AKT FW | Tsingke | TCCTCCTCAAGAATGATGGCA |
| AKT REV | Tsingke | GTGCGTTCGATGACAGTGGT |
| APC FW | Sangon | GAGGAATTTGTCTTGGCGAG |
| APC REV | Sangon | TGTTTGTCTGGCTCCGGTAA |
| APC2 FW | Tsingke | ATGACCCTCACCAACCTCACCT |
| APC2 REV | Tsingke | GCCTCCCTCAGCACCTTCTTG |
| AXIN FW | Tsingke | CAGAAAATCATGCAGTGGATCATT |
| AXIN REV | Tsingke | GATGAAGAGGTGGGAGGGCT |
| CCND1 FW | Sangon | AAGTGCGAGGAGGAGGTCTT |
| CCND1 REV | Sangon | GCGTGTTTGCGGATGATCT |
| CDH1 FW | Sangon | CACAGCAGAACTAACACACGG |
| CDH1 REV | Sangon | CAGCAAGAGCAGCAGAATCAG |
| CTNNB1 FW | Tsingke | CATCTACACAGTTTGATGCTGCT |
| CTNNB1 REV | Tsingke | GCAGTTTTGTCAGTTCAGGGA |
| CTNNBIP1 FW | Tsingke | TACATTCAGCAGAAGGTCCGAGT |
| CTNNBIP1 REV | Tsingke | CCTCTGCACCCTGGTCGAT |
| DVL1 FW | Tsingke | TGAGTCCAGCAGCTTTGTGGA |
| DVL1 REV | Tsingke | ATGCTGATGCCCAGAAAGTGAT |
| FZD10 FW | Tsingke | ACGTGTACTGGAGCCGCGA |
| FZD10 REV | Tsingke | GCGAAGAGGCGGATGAGGTA |
| ILK FW | Tsingke | TGGAACCCTGAACAAACACTC |
| ILK REV | Tsingke | AGCACCTTCACGACAATGTCA |
| JUN FW | Tsingke | CTGAAGGAGGAGCCTCAGACAGT |
| JUN REV | Tsingke | CTGTTTAAGCTGTGCCACCTGTT |
| LEF1 FW | Tsingke | TGCCAAATATGAATAACGACCCA |
| LEF1 REV | Tsingke | GAGAAAAGTGCTCGTCACTGT |
| MET FW | Sangon | ATGTGTGGTCCTTTGGCGT |
| MET REV | Sangon | TCTGGGCAGTATTCGGGTT |
| MGAT5 FW | Sangon | CACTTTACCATCCAGCAGCGA |
| MGAT5 REV | Sangon | GGTTGAGTTTGTTCCGGTGC |
| MMP10 FW | Tsingke | TCAGTCTCTCTACGGACCTCC |
| MMP10 REV | Tsingke | CAGTGGGATCTTCGCCAAAAATA |
| MMP2 FW | Tsingke | ACGGAAAGATGTGGTGTGCG |
| MMP2 REV | Tsingke | CAAGGTCAATGTCAGGAGAGGC |
| MMP9 FW | Tsingke | TCGACGATGACGAGTTGTGG |
| MMP9 REV | Tsingke | GGCCTTGGAAGATGAATGGA |
| MTA1 FW | Tsingke | TGGAGAATCCGGAAATGGT |
| MTA1 REV | Tsingke | GCTGTGGGTCGTAGACTAGAGAA |
| MYC FW | Tsingke | AAGATGAGGAAGAAATCGATGTTGT |
| MYC REV | Tsingke | TGATGTGTGGAGACGTGGCA |
| Nrf1 FW | Tsingke | GCTGGACACCATCCTGAATC |
| Nrf1 REV | Tsingke | CCTTCTGCTTCATCTGTCGC |
| OGT FW | Sangon | GGCAGTTCGCTTGTATCGT |
| OGT REV | Sangon | GATGGCACGCGTATAACAC |
| p53 FW | Tsingke | CAGCACATGACGGAGGTTGT |
| p53 REV | Tsingke | TCATCCAAATACTCCACACGC |
| PDK1 FW | Tsingke | GGAACAGCGCAGTACGTTTCT |
| PDK1 REV | Tsingke | CTCGTTTCCAGCTCGGAATGG |
| PI3KCα FW | Tsingke | CCACGACCATCATCAGGTGAA |
| PI3KCα REV | Tsingke | CCTCACGGAGGCATTCTAAAGT |
| PI3KCβ FW | Tsingke | TATTTGGACTTTGCGACAAGACT |
| PI3KCβ REV | Tsingke | TCGAACGTACTGGTCTGGATAG |
| PTEN FW | Tsingke | TTTGAAGACCATAACCCACCAC |
| PTEN REV | Tsingke | ATTACACCAGTTCGTCCCTTTC |
| SMAD4 FW | Tsingke | GCCCAGGATCAGTAGGTGGAATA |
| SMAD4 REV | Tsingke | TGCAATCGGCATGGTATGAAGT |
| TCF4 FW | Tsingke | GCCTCTTATCACGTACAGCAAT |
| TCF4 REV | Tsingke | GCCAGGCGATAGTGGGTAAT |
| VEGFA FW | Sangon | TCGCTTACTCTCACCTGCTTCT |
| VEGFA REV | Sangon | CAACCACTCACACACACACAAC |
| WNT11 FW | Tsingke | GTGAAGGACTCGGAACTCGTCTAT |
| WNT11 REV | Tsingke | CGTAGCAGCACCAGTGGTACTTA |
| WNT5A FW | Tsingke | CAATTCTTGGTGGTCGCTAGGTA |
| WNT5A REV | Tsingke | TACTGCATGTGGTCCTGATACAAGT |
| WNT7A FW | Tsingke | CACGGACCTGGTGTACATCGA |
| WNT7A REV | Tsingke | TGACATAGCAGCACCAGTGGAA |
| β-actin FW | Tsingke | CATGTACGTTGCTATCCAGGC |
| β-actin REV | Tsingke | CTCCTTAATGTCACGCACGAT |
| **Oligonucleotides for construct** |  |  |
| CDH1-ARE1 FW | Tsingke | CACCTAGGGAATCAATTTGCTGACTCACTAACCCATGAAGCTCTA |
| CDH1-ARE1 REV | Tsingke | GATCTAGAGCTTCATGGGTTAGTGAGTCAGCAAATTGATTCCCTAGGTGGTAC |
| CDH1-ARE1m FW | Tsingke | CACCTAGGGAATCAATTTTTTGACTCACTAACCCATGAAGCTCTA |
| CDH1-ARE1m REV | Tsingke | GATCTAGAGCTTCATGGGTTAGTGAGTCAAAAAATTGATTCCCTAGGTGGTAC |
| FZD10-ARE FW | Tsingke | CCCCGCCGCTGCTTTGCATGAGAAAGCGCAGCGGCCCGGGGCAGA |
| FZD10-ARE REV | Tsingke | GATCTCTGCCCCGGGCCGCTGCGCTTTCTCATGCAAAGCAGCGGCGGGGGTAC |
| FZD10-AREm FW | Tsingke | CCCCGCCGCTGCTTTGCATTCGAAAATGCAGCGGCCCGGGGCAGA |
| FZD10-AREm REV | Tsingke | GATCTCTGCCCCGGGCCGCTGCATTTTCGAATGCAAAGCAGCGGCGGGGGTAC |
| FZD10-P FW | Tsingke | GGAAGATCTTGCCCGGAGTTTCTACCTTCTTTTT |
| FZD10-P REV | Tsingke | CTAGCTAGCGGGATGAGAAGACTCGCAAAAAGGC |
| JUN-ARE FW | Tsingke | CGGAGAATGTTCTCTCCTTGAGGAAGCAACTGGATCTTGTCATCA |
| JUN-ARE REV | Tsingke | GATCTGATGACAAGATCCAGTTGCTTCCTCAAGGAGAGAACATTCTCCGGTAC |
| JUN-AREm FW | Tsingke | CGGAGAATGTTCTCTCCTTTCGGAAATAACTGGATCTTGTCATCA |
| JUN-AREm REV | Tsingke | GATCTGATGACAAGATCCAGTTATTTCCGAAAGGAGAGAACATTCTCCGGTAC |
| JUN-P FW | Tsingke | GGGGTACCCCGCAACTCCCTGAATACAACAGAAAATGATTC |
| JUN-P REV | Tsingke | CTAGCTAGCTAGCCACTCCCGCCTCGCTGCTTCAGCCACACT |
| LEF1-ARE1 FW | Tsingke | CATGGGGCTATAAATGTCTGACACAGCTACACGTTCACCTCTCTA |
| LEF1-ARE1 REV | Tsingke | GATCTAGAGAGGTGAACGTGTAGCTGTGTCAGACATTTATAGCCCCATGGTAC |
| LEF1-ARE1m FW | Tsingke | CATGGGGCTATAAATGTCTTCCACAATTACACGTTCACCTCTCTA |
| LEF1-ARE1m REV | Tsingke | GATCTAGAGAGGTGAACGTGTAATTGTGGAAGACATTTATAGCCCCATGGTAC |
| LEF1-ARE2 FW | Tsingke | CAATCCATTTTTAATAGCTGACAATGCTATGCCTCAAGAGAAGAA |
| LEF1-ARE2 REV | Tsingke | GATCTTCTTCTCTTGAGGCATAGCATTGTCAGCTATTAAAAATGGATTGGTAC |
| LEF1-ARE2m FW | Tsingke | CAATCCATTTTTAATAGCTTCCAATATTATGCCTCAAGAGAAGAA |
| LEF1-ARE2m REV | Tsingke | GATCTTCTTCTCTTGAGGCATAATATTGGAAGCTATTAAAAATGGATTGGTAC |
| LEF1-P FW | Tsingke | GGAAGATCTTCCATTCTGTGTTCTCCCCTCCCCCTCCTGAGG |
| LEF1-P REV | Tsingke | CTAGCTAGCTAGGTGCGAGGCTCCGGGCGCGTCCTGGTTCCT |
| MMP9-ARE2 FW | Tsingke | CACACACACCCTGACCCCTGAGTCAGCACTTGCCTGTCAAGGAGA |
| MMP9-ARE2 REV | Tsingke | GATCTCTCCTTGACAGGCAAGTGCTGACTCAGGGGTCAGGGTGTGTGTGGTAC |
| MMP9-ARE2m FW | Tsingke | CACACACACCCTGACCCCTGAGTCAAAACTTGCCTGTCAAGGAGA |
| MMP9-ARE2m REV | Tsingke | GATCTCTCCTTGACAGGCAAGTTTTGACTCAGGGGTCAGGGTGTGTGTGGTAC |
| p53-ARE1 FW | Tsingke | CAGCCAAGTCTGTGACTTGCACGGTCAGTTGCCCTGAGGGGCTGA |
| p53-ARE1 REV | Tsingke | GATCTCAGCCCCTCAGGGCAACTGACCGTGCAAGTCACAGACTTGGCTGGTAC |
| p53-ARE1m FW | Tsingke | CAGCCAAGTCTGTGACTTTTACGGTCAGTTGCCCTGAGGGGCTGA |
| p53-ARE1m REV | Tsingke | GATCTCAGCCCCTCAGGGCAACTGACCGTAAAAGTCACAGACTTGGCTGGTAC |
| PDGFB-ARE1 FW | Tsingke | CCACCTACTTTTTTTTTTGCCTCGTCAGCCCGACGCCCCTCAAAA |
| PDGFB-ARE1 REV | Tsingke | GATCTTTTGAGGGGCGTCGGGCTGACGAGGCAAAAAAAAAAGTAGGTGGGTAC |
| PDGFB-ARE1m FW | Tsingke | CCACCTACTTTTTTTTTTTTCTCGTCAGCCCGACGCCCCTCAAAA |
| PDGFB-ARE1m REV | Tsingke | GATCTTTTGAGGGGCGTCGGGCTGACGAGAAAAAAAAAAAAGTAGGTGGGTAC |
| PTEN-ARE1 FW | Tsingke | CTCCCTCTACACTGAGCAGCGTGGTCACCTGGTCCTTTTCACCTA |
| PTEN-ARE1 REV | Tsingke | GATCTAGGTGAAAAGGACCAGGTGACCACGCTGCTCAGTGTAGAGGGAGGTAC |
| PTEN-ARE1m FW | Tsingke | CTCCCTCTACACTGAGCATTGTGGTCACCTGGTCCTTTTCACCTA |
| PTEN-ARE1m REV | Tsingke | GATCTAGGTGAAAAGGACCAGGTGACCACAATGCTCAGTGTAGAGGGAGGTAC |
| PTEN-ARE2 FW | Tsingke | CCCTCAGACTCGAGTCAGTGACACTGCTCAACGCACCCATCTCAA |
| PTEN-ARE2 REV | Tsingke | GATCTTGAGATGGGTGCGTTGAGCAGTGTCACTGACTCGAGTCTGAGGGGTAC |
| PTEN-ARE2m FW | Tsingke | CCCTCAGACTCGAGTCAGTGACACTAATCAACGCACCCATCTCAA |
| PTEN-ARE2m REV | Tsingke | GATCTTGAGATGGGTGCGTTGATTAGTGTCACTGACTCGAGTCTGAGGGGTAC |
| SMAD4-AP1 FW | Tsingke | CAGCCATGCCTGGAATCCTGACTCAGAGAATCTGTAAGATCAA |
| SMAD4-AP1 REV | Tsingke | GATCTTGATCTTACAGATTCTCTGAGTCAGGATTCCAGGCATGGCTGGTAC |
| SMAD4-AP1m FW | Tsingke | CAGCCATGCCTGGAATCCTTCCCAGGAGAATCTGTAAGATCAA |
| SMAD4-AP1m REV | Tsingke | GATCTTGATCTTACAGATTCTCCTGGGAAGGATTCCAGGCATGGCTGGTAC |
| SMAD4-ARElike FW | Tsingke | CTGATCTTACAGATTCTCTGAGTCAGGATTCCAGGCATGGCTA |
| SMAD4-ARElike REV | Tsingke | GATCTAGCCATGCCTGGAATCCTGACTCAGAGAATCTGTAAGATCAGGTAC |
| SMAD4-ARElikem FW | Tsingke | CTGATCTTACAGATTCTCTTCGCAGGGATTCCAGGCATGGCTA |
| SMAD4-ARE-likem REV | Tsingke | GATCTAGCCATGCCTGGAATCCCTGCGAAGAGAATCTGTAAGATCAGGTAC |
| SMAD4-P FW | Tsingke | CGGGGTACCCCGGACGGTGAAACCTACAGGTTTAAGG |
| SMAD4-P REV | Tsingke | CTAGCTAGCTAGGTAGAGTGGGCGTCCAGTAAGTGTT |
| TCF4-ARE FW | Tsingke | CAATAATGATACCGAGATTGACAAGGCCAACGAATTCCCCTGCAA |
| TCF4-ARE REV | Tsingke | GATCTTGCAGGGGAATTCGTTGGCCTTGTCAATCTCGGTATCATTATTGGTAC |
| TCF4-AREm FW | Tsingke | CAATAATGATACCGAGATTTCCAAGATCAACGAATTCCCCTGCAA |
| TCF4-AREm REV | Tsingke | GATCTTGCAGGGGAATTCGTTGATCTTGGAAATCTCGGTATCATTATTGGTAC |
| TCF4-P FW | Tsingke | GGAAGATCTTCCCTGACGTTGGAGGTAGTAGGAAATGAGC |
| TCF4-P REV | Tsingke | CTAGCTAGCTAGTTTGCCTGCATCTTATTCTTTAGTG |
| VAV1-ARE1 FW | Tsingke | CCGGAGCTTGCAGTGAACTGAGATTGCGCCACCGCACTCCAGCCA |
| VAV1-ARE1 REV | Tsingke | GATCTGGCTGGAGTGCGGTGGCGCAATCTCAGTTCACTGCAAGCTCCGGGTAC |
| VAV1-ARE1m FW | Tsingke | CCGGAGCTTGCAGTGAACTGAGATTAAGCCACCGCACTCCAGCCA |
| VAV1-ARE1m REV | Tsingke | GATCTGGCTGGAGTGCGGTGGCTTAATCTCAGTTCACTGCAAGCTCCGGGTAC |
| VAV1-ARE3 FW | Tsingke | CGAAGACCAGCTGAGTGATGACGGGGCTGGACCAGACAGAGGAGA |
| VAV1-ARE3 REV | Tsingke | GATCTCTCCTCTGTCTGGTCCAGCCCCGTCATCACTCAGCTGGTCTTCGGTAC |
| VAV1-ARE3m FW | Tsingke | CGAAGACCAGCTGAGTGATGACGGGAATGGACCAGACAGAGGAGA |
| VAV1-ARE3m REV | Tsingke | GATCTCTCCTCTGTCTGGTCCATTCCCGTCATCACTCAGCTGGTCTTCGGTAC |
| WNT11-ARE1 FW | Tsingke | CACAACCCGTCTCCCGGGTGACCCGGCGCCGCGTGCGCAGCCAAA |
| WNT11-ARE1 REV | Tsingke | GATCTTTGGCTGCGCACGCGGCGCCGGGTCACCCGGGAGACGGGTTGTGGTAC |
| WNT11-ARE1m FW | Tsingke | CACAACCCGTCTCCCGGGTTCCCCGATGCCGCGTGCGCAGCCAAA |
| WNT11-ARE1m REV | Tsingke | GATCTTTGGCTGCGCACGCGGCATCGGGGAACCCGGGAGACGGGTTGTGGTAC |
| WNT11-ARE2 FW | Tsingke | CACACAGATCCCCCGCTGTGAGTCCGCGCGCCTCCGTCCTCTTGA |
| WNT11-ARE2 REV | Tsingke | GATCTCAAGAGGACGGAGGCGCGCGGACTCACAGCGGGGGATCTGTGTGGTAC |
| WNT11-ARE2m FW | Tsingke | CACACAGATCCCCCGCTGTTCGTCCATGCGCCTCCGTCCTCTTGA |
| WNT11-ARE2m REV | Tsingke | GATCTCAAGAGGACGGAGGCGCATGGACGAACAGCGGGGGATCTGTGTGGTAC |
| WNT11-ARE3 FW | Tsingke | CTTTCCTCATCTGTGAGATGAGGCAGCGATAGTGCCTATCTCACA |
| WNT11-ARE3 REV | Tsingke | GATCTGTGAGATAGGCACTATCGCTGCCTCATCTCACAGATGAGGAAAGGTAC |
| WNT11-ARE3m FW | Tsingke | CTTTCCTCATCTGTGAGATTCGGCAATGATAGTGCCTATCTCACA |
| WNT11-ARE3m REV | Tsingke | GATCTGTGAGATAGGCACTATCATTGCCGAATCTCACAGATGAGGAAAGGTAC |
| WNT11-ARE4 FW | Tsingke | CCACGCAGCTCACGGAGCTGACCCCGCCAACCAGGGACGCCGAGA |
| WNT11-ARE4 REV | Tsingke | GATCTCTCGGCGTCCCTGGTTGGCGGGGTCAGCTCCGTGAGCTGCGTGGGTAC |
| WNT11-ARE4m FW | Tsingke | CACGCAGCTCACGGAGCTTCCCCCATCAACCAGGGACGCCGAGA |
| WNT11-ARE4m REV | Tsingke | GATCTCTCGGCGTCCCTGGTTGATGGGGGAAGCTCCGTGAGCTGCGTGGGTAC |
| WNT11-ARE5 FW | Tsingke | CCGGGGTCACCTTCCGCCTGAGCTCGCGGGTCAGGGTGCACGCGA |
| WNT11-ARE5 REV | Tsingke | GATCTCGCGTGCACCCTGACCCGCGAGCTCAGGCGGAAGGTGACCCCGGGTAC |
| WNT11-ARE5m FW | Tsingke | CCGGGGTCACCTTCCGCCTTCGCTCATGGGTCAGGGTGCACGCGA |
| WNT11-ARE5m REV | Tsingke | GATCTCGCGTGCACCCTGACCCATGAGCGAAGGCGGAAGGTGACCCCGGGTAC |
| WNT11-P FW | Tsingke | CGGGGTACCGTTTACTGACCTGAGCTCCAGGCTG |
| WNT11-P REV | Tsingke | GGAAGATCTGCTCACGCCTGTAATCCCAGCACTTTGG |
| **Recombinant DNA** |  |  |
| pARE-luc | Zhang’s | ([1](#_ENREF_1)) |
| pcDNA3.1 | Invitrogen | V79020 |
| pGL3-Basic | Promega | VQP0121 |
| pGL3-promoter | Promega | VQP0124 |
| pRL-TK | Promega | VQP0126 |
| **Software and Algorithms** |  |  |
| Canvas 9 | Cancas GFX, Inc. | https://www.canvasgfx.com/ |
| Cytoscape | N/A | http://www.cytoscape.org/ |
| Excel | Microsoft | https://www.microsoft.com/ |
| FlowJo 7.6.5 | FlowJo | https://www.flowjo.com/ |
| IGV | N/A | http://www.igv.org |
| Image J | N/A | https://imagej.nih.gov/ij/ |
| KEGG | Kanehisa Laboratories | https://www.kegg.jp/ |

1. Zhang, Y., and Hayes, J. D. (2010) Identification of topological determinants in the N-terminal domain of transcription factor Nrf1 that control its orientation in the endoplasmic reticulum membrane. *The Biochemical journal* **430**, 497-510
