# Supplementary material for: Nrf1 Is Endowed with a Dominant Tumor-Repressing Effect onto the Wnt/β-Catenin-Dependent and Wnt/β-Catenin-Independent Signaling Networks in the Human Liver Cancer
Source: Oxid Med Cell Longev. 2020 Mar 23;2020:5138539. doi: 10.1155/2020/5138539 (PMC7125503; doi:10.1155/2020/5138539)
Supplement: Supplementary 2 — Table S1: the key resources used in this work. Table S2: the sequencing data of genes encoding proteasomal subunits. Table S3: the sequencing data of genes involved in Wnt/β-catenin signaling pathway. Table S4: the promoters, the enhancer ARE/AP1-binding sequences, and the corresponding mutation sequences of the representative genes of Wnt/β-catenin signaling components. Table S5: the sequencing data of genes involved in the interactive network of Nrf1 interactors, migration and invasion pathways, carcinoma related pathways, signal transduction pathways, and metabolism pathways. Table S6: the sequencing data of DEGs whose RPKM values are greater than 3 in at least one cell line (shNC- or shNrf1-HepG2). Table S7: the sequencing data of genes implicated in the focal adhesion and ECM-receptor interaction. Table S8: the sequencing data of genes responsible for the pathways involved in cancer. Table S9: the promoters, the enhancer ARE-binding sequences, and the corresponding mutation sequences of PTEN, p53, CDH1, VAV1, PDGFB, and MMP9. [file 5138539.f2.zip › Table S4_ARE- or AP1-binding sequences.docx]

| **FZD10** | CGCAAAAAGGCAAAGGGGGGCAGAAAAGCCGAGCGCTGACAGGCCCCGCCCCCGGGCCGCGCGCCCCGC**CCCGCCGCTGCTTTGCATGAGAAAGCGCAGCGGCCCGGGGCAG**CCTCCTCC  **Mut: TAGGAAAAT**  CCGCCGCCCCGCCGCGGGCTCCCGGGCCCCGCGGGGGCCGATCCCCCCCGGCCTGCAGCCCAGCCTGCGGGGGGCGCAGCGCCTAGTCCCGCCCGCGTCGCCCGCCAGCCCCGGGACAGCCCGCCTGGCGCGCGGGGCTCTGCGCGCTGCGACCCTCCCCGGCAGTCCCGCCCGCCTCTCCTCGCCCTCTCTCTGCCTGGCCTCCAACTTTGCCTCTCGCTATCCTCTCCGCCGGCGCCCCCTTTCCCGGCGGTCCCCGGGATGGTCCCCGGCACCCCGAGGACGCCGAGGTTACGGGAAGTTCGGGGACTGGCGGGAGAAGGGGTTTGGGGGTGGTCCGCGGCGAGGGGCGCGGGGTGCATCTTCTCTTCCTTTTATGGTGAGTCCCGGGCGGAGGGCGCTTTCAAAGGGAGGTGGGATTTGCGTTTTATAGCCTGGGCCATAAAGTAGGCCCTTGCTTGTAAACCTGCCCGCATCTTCCTGAGGCTGGGGAGGTGCTACCAGCCCCGGGCTCCGGGCTCCGAGGAGGGATTTTCTTTGAAATTTCAAAGAGACGAGCTAAGGGGAGGGGGAGGGGCTCGAGAATCCGGATTTTAGGGCCGCTCATCCGAGGTCTCCTCTAAGAAGGCGGGAAGGGGGAGGGGCGGGAAGCGTCCCGGGACTTTGAACACCGCCTCCCACCCCGCGGGAAGTGCGGGCTTGGTTTGTACCGCGGTGACCCCCGCCCCCTCCGAAGCCGCAGAGCCGGGGCCTCGCGCCAGCAGGGCTGGAGATGCCTTCTTGGCGGCTGAGTTTATTTATTATAGGAAGTCATTCGCTCGTGGGGTATTTATGTGATTTGGCGAGTGATGTGCCCGGCCAGCGCCCTCCTTGGCTGCAGCCCCGCAGGAGGACCCGGAGTAGGGTGGGATGGAGTGGGTCGTGGGAGGAGCGCGTCAGCGCCTGCCCGGGGACCCCCAGCTCCCGCGAGGACACGGAGGCGCGCACGCCGCTCGGTTTTCCTGGAAAGTGGAGAAGGAGCGTCCTGGGCAGGTCCTCTGAGCTCATCCCCCCTCGGATTGGGGCGGGTCTGTGACGGGGTCACTTAGGACACGACGTCCCCCCGCCATTCCCTTCCCCCGCCCAGGGCGTTCGCGGTGGGCGCCCACCGCCAAGCCCCACTGTTCCCAAGGATGCGCCAGGTGCTTCCCGTAGCGTCCTGGGTTGACCCTTAAAAAAACAGCACCCCTAGGAGGTGGCCGGCCCTCTCCTCCCAGGGTCTCTCCGGGTCACGATCTTCCAAAGTTCGGAAACTCGCAGGATCGCGTGTGCAATCTCCCGCTACCTCCCGGGGGGCCGGGGAGAGGTCAGAGGAGCGAGTCCCGCGTCCACCGGCCTCGCTTGCCCCCTGCCCGTTTGAGGATAGTTCCAGGGAGCAGGGTGGAGTGTGCGGACATCTTTGGAGGCAGTGCTGGGGCTTCCCGCGTTGGCGGCGCTCCACCCGGCGTGGGGGGCGGCTGCACGGGCCCCCGCGGTGGGGACGCTGCGCACGGGGCAAGGTCTCCCTAGGAAGCGCCCGGGAAGGAGATGGGGCCCGCCAGGAACCCCCCTCACTGACCAGCTTTCTGCACGCCGTGCAGGAGGGGGCCACTTCCTCGGAGAGTATTGGCTTTTAATTAAAACAAGCCCTACAATTTTTACATCGGTAAGAAACTTGGGGAAATCTCACCTTTCCACTCATTCATTCATCCGTTCAACAGACATTTGAGCGCCCAGAATCCCAGAATTAATAGGCAGGTTCAGGCACCCACCCCTGGGTCGCTGTCCCTGGGTGTCCTGGGTCTCACTAGGAGGTGCTGGAGGAAGTCGGGGTGAGGGAGAAAGTGAAAAAGAAGGTAGAAACTCCGGGCA |
| --- | --- |
| **JUN** | CAACTCCCTGAATACAACAGAAAATGATTCAGGGCAACAGACAGA**GGAGAATGTTCTCTCCTTGAGGAAGCAACTGGATCTTGTCATC**ACTGTATACCTACCTACCCCACCCCCTCCCCA  **TAGGGAAAT :Mut**  GCTCAGTGCCTGGCTCACAGTAGGCTTTCAGTTACCCTCTGCAGATCAGTGAAAGCTAGGTGAGTGCCCGGAGTGAAGAAAAGTTGGCAGGTTTCCCACTGATACCAGCTGCTGTTGGTTTCTGAACACTCAAAGCCGCAAATACCTTAGGGCTGGGGGCAATGAACCCAAGGCTGAATTCCAAGTTCAGAAGCAGCGAAGTCTGAATTTAGAACCTAGGAACTTAAACTGCTGCAGGTCCAACTTCAAGCCCCAGTTTTAGACAGAGGCTTGGGAAAGATCTGACTTCTAACCCGGTTCCCCCTCCCCTCCTCCCCTCGATGCTTCTCACAGGAAAGTACACCTGGTCCTGCCAAATCGCACTCTTATATCCTGGCATCCTATCCAGGCTCTGCGAGGATGGAAACTGCGAGGCAGGGGAGGGAAGCGGGCTGTTTGGCCACCACCTCCCTAGTGCTGCAGGCGACCCTGTCACACTAACTCCTGGCAGCCCAGTGAGGTGGACGGCACCGGCCCCACCTGCAGATGAGGGAAATGAAGCTCGGAGGAGTTCCGTGATTTGCTTGCTTCACACTGTGGTAGCCTGGCCACGAAAGAACCAGGATTCCCGACTTCGGGATTCTTTCCACCACACACTTTCGTCCCTAAGGGGTGGGGGGCGGGGGGAGAATAAAATAACCGCGGAAAAGGAACCACTTACATGTGTCTAGCGCTTCCTAGAGGCTACCCAGGATATGCGCCCACCACCCGGCCGGGAGTGCAGAGATTTGAAGTCCAGGTTCTACCCCGGGCTCCGAGTACTACTGCGTGACTTTATGCGAGTGTCCGCCGCCTTCTGGGCTTGTTTTCCCGGAAGCAACTCGGCGCGGATGGAGTGTGTGTGTGCGCGCGCGCGCGCGTTATGTTGTGCGTGTTGTGTTAAGCGTGTGCGTGTTGTCCGGGGGCGGGAGGGGGAGTAGACTAACACCGGGGTTCCCCGAGTTTCGGATCGCCTACACGCTTGTTCCCATCTGGACCCTGTTACCCACCAATTGCGCCCACTATAAAAACTGCCCCTCCGAGGCAAAGCTGTGAACCCCCGCGCCCTTTCCCCCACGGTCCCGGAGGATGAAGTGGGGTGCAACGGAGACTCAGCTGAGCGTCCAGTTTCGGGCAATACAAATCTCTCGGCTTCTACGAGCAGCCAGACGACCCCGCGGACCGTCGCTCCTGAACTTGACCGAGATGCAAACTTCGGAGTGTTCTCAACGTGGGGGGCCGACTCTCGGGAGACCGCCCCTAAACTTAAGTCCCCTTAGGCTCGCCCCCACCTGGGACTTCACAGAGCCACCTTAAGGGCGGTATTCCCGCCCCCCCGGAAGTGCGGGGGGGTGGCAGCGTACTTGGATTCTCAGCCTCCAGCCCCGCGCGGTGGCGGCCGCCGGTGGATGACTTCGGGCCCCACAAGTGGGGAAACAACAACCACCCCTCGCCCGCACCCCTGGCCCAAAACAACTGGCCAGGTTCCCTGGCCTCCCGGGTCCCTGCATCCCCCGCATCCCCGTCCGCAGCCGTGAACTTGAGCCCCCCTCCATCAGAGGTTGCGAGCGTCCGCCCGCTCGCGGCAGCCACCGTCACTAGACAGTCAAACCCCAAGACGTCAGCCCACAATGCACCGGGCGGGCCGGGAAAAACGGCCCGGGGAGGGGACCGGGGAAGAGAGGGCCGAGAGGCGTGCGGCAGGGGGGAGGGTAGGAGAAAGAAGGGCCCGACTGTAGGAGGGCAGCGGAGCATTACCTCATCCCGTGAGCCTCCGCGGGCCCAGAGAAGAATCTTCTAGGGTGGAGTCTCCATGGTGACGGGCGGGCCCGCCCCCCTGAGAGCGACGCGAGCCAATGGGAAGGCCTTGGGGTGACATCATGGGCTATTTTTAGGGGTTGACTGGTAGCAGATAAGTGTTGAGCTCGGGCTGGATAAGGGCTCAGAGTTGCACTGAGTGTGGCTGAAGCAGCGAGGCGGGAGTGG |
| **LEF1** | ATTCTGTGTTCTCCCCTCCCCCTCCTGAGGGTTTGATGAAAAGGAACCTCTACTCTGCTTTGGTGAAACTGCATTTTCCACTTGTTTTCCTATTGGTCAAATTGGTGCAGTTATGGGAGGACTTTTGACTGTTTTCCCATGTGTCTTTTAAGA**ATGGGGCTATAAATGTCTGACACAGCT**  **Mut(ARE1): TAGCACAAT**  **ACACGTTCACCTCTCT**GGGACACTACTCACACTGGTGCCTAACTTTAAGGGAAGTGACATACCACCATCATCCAAAAACAAAAATTGGGTTTCTCACACACTGTAGCAGAATATACTCTTTT**TCTTCTCTTGAGGCATAGCATTGTCAGCTATTAAAAATGGATT**TAATGTTTTAATTTT  **Mut(ARE2): ATATTGCTA**  CCTGGATTCCTTCACCAACCCCTTAATTTCACCCCAATCTTCCAGATAGAATTTACATGTGACTACTGGCCATGTTGTAGGGACCAATTGGATCCTTTGTAAAGTTTTAAAAATTGCTTCCTAGTAAATTCTTATTCCCAAGAAGTAGTCGTTATTAGTAGGTGAAGTCATTAGTGCTCACTTAGATACAGCATTGTAAAAATCTTTACCCATTCAATGTTCAGCAGCCTGAAATTTCACCTTTAGGAAAAAAAGAGGGATCTTTGCCAGACAATTCCTATATAATGGAGCAAAATCTGATTCTGTAGTGTTTTGCTTGTTTTCGATTTAAGGGAGATGAAGATACATTTCTTTATGTCCTTTGTTTACTGTTCTGAAGTTTTACAGATGGTTACTGTATTGGATATAATAATTTTGCCTAGATTTTTAACTGAGGGGGTTTGAGAACCAAGGGACAAAATGTAATGTGTTTTCAAACTTCAGCTTCCCTTCTGCTGTAACTTTCACAAAGTATCTCCTGAAAACTCCTGTCTTTGTACAAAATTCATCAAAGAGACAGTGGAGACTGGGGAATTTTTGAGGTTGTATTCGGATTGGTGTCTTCAAGGGTCCCTTTCAGTTCGCAGTCCAAGGTGCCAGGGACTACCCCGTCCTCCCTCCCTCTTCTGGCTTTGCTCTCCTGAGTCCTTTTGCCTTCTCTTCCCCCTTCTTGGATTATCTTTTCATTCTCGATGAGGTTCCCACACACTGCGGTGTGTGTCTCTGAAAACCCACTGGAGACCTAGCACAACTCTCCGTACATCCCGTGGTGAGAACAGAATGAAAGATATATTGTTTAAAAAGCAATAATTAAAATCTAGTCTTCAGTTCCTTCTTCCTCGTCATATTTTTTCTCCGTAAGCCTAGAGATTTTATTTTCACTAGTGTGTTCCCTTGTCTCCAAAGAGCGTGTGTGTGATATTATATTCGGGAAAGCTACAACTCTCTTTTCCTTGTCCTTCTGTTCTCTCTTAATAGTTGAGCAATGTCTGTTATATTTTCCCCTTTTCCTTTTTTTCTCAGTCCCAGATTCCCGCCTCTCCCCACTGTCAGAGCATCTATCAATGTGGTGTCCATCACAGCGGCAGCGGCTTTCTCTTTCATCTTCCTCCCTCTGCCAGAGCCAGGGAGGGAGAGTGGGAGGCGTCAAGGAGGTAGGGGAGAGACTGGCAGAGGAAAAGGAGTGGGTGGGTGGGGGCCAAGTAAATAGATACTTAGATGATGAAGTCAAGCCACTGCGGCAATGTTTCTTGTCAGTTTCACGCGGGCAAAGCGTGCCTTTCGGTGGGTTATAAGCAGCGCCCGGTCCTTCCTTCTCTCGCCAAGTTGCCTGATCCTTCCCTCCAGGCGCGCGCGCACACACCACACTCACACACCCCAAAACCAAGACTCGTCCTACAGGATCTGGGAAAAGAAAAAGAAAAAAAAGCCCTCAATCACCACCTCCTTCTCGCCGACTCCCCCTCACCCCCCGCCTCCCCTCCAGCGGGCAGCCAAGGAGAGCTAGAGGCGGGGGAGGGGAGAGGGAGGAGAAGCGACGCAAGTGGGTAGCTTTTCAGCGCCGGCGAGGCGCGGGAGGAGGAGAAGCAGTGGGGAGGCGCAGCCGCTCACCTGCGGGGCAGGGCGCGGAGGAGGGACCCGGGCTGCGCGCTCTCGGGCCGAGGAACCAGGACGCGCCCGGAGCCTCGCAC |
| **SMAD4** | GCTGACGGTGAAACCTACAGGTTTAAGGGCTTAAATCTCAACCTTTGTGTTAGGAGTAACAGGAGTGTGCTGAGAGGGCAAGCAATAAAACAAGTCATACCAAAAGGCCACATTGGTCTCTCCTAAGCCCCAATCCCACTCCACTCCTGTGGCCAGTAGTCCAAACAGAAAATAACTGGAGAAGACGAGGAGGTCAAGGATCAGGAAACTAAACGTTATGTGAATTCACCAGCAAGATGTACAGAACGCTTGCGTTTACATTGTTTTTATGGAACTAGCAGAATAAAACTGATCTATTTTAAAAATGAAAAAAAAAAAAAAACAAAAGAAAGAAAACACAGTTGCCACTACTTGAGAGGTGGAAGGAGGAGCAGTGTCCCAGGGCAAAGTTGGGTTTCCTTAAGTAAAAAGGTGAAGGGAACCTGAAGGGAAATTCTGAGAAGAGGTTGAGCTTACAGGGAATATCATTTGCATAAATAAATACATGGTGTGGTAGACTCTAAAGAGGCCCCATATGATTCCCACCTCCTGGTGTTCAAGCTTTTGTGTGTTTCCTTCCCCTTCAATGTAGGTGGCGCCTGTGATTTGCTTCTGGCCAGCAATGTATGGCAAAGGTGACAGAGTATGAGTGATACTGTGTATGTGATGATGTTACATAAGATTGAAACCAGTGTCTTGCTGAGACTCTTCCCGTTGCTGGCTTCGAAGAGGCAAGCTGCCATATTGTTAGCTTCCCTATGGAGAGCACCACATGGCAAGGAACAGAGGGGGTCTCCAGCCAACAGCCTGCAAAGGACTAAGGCCCTCAGTCCTGTAGCCTGCAAGGAACTGAATGGTGCCAACAACCACATGAACTTGGAAGCAGATCCTTTGTTCCAGCCTCACTTTGTAGAGGACCCA  **Mut(ARE-like):3′-GGCAGGCTT-5′**  3′-**TCGGTACGGACCTTAGG*ACTGAGT*CTCTTAGACATTCTAGT**-5′  GCTA**AGCCATGCCTGGAATCCTGACTCAGAGAATCTGTAAGATCA**TAAATGTTTGTTGTT  **Mut(AP-1): 5′-TTCCGAC-3′**  TTAGGCTTCTATATTTGTGGTGAGATTGTTATACAGCAATAGGTAACTAATACAGACGGAATAGAAGAAGTTTAGTTATAACAATGTCTCAATTTCTTAAAAACTTCTTCTGAGGTATATTACCTAAGATTCACATATTGATCTGTTACCAAATCATGTTTTGTAATCTATCAGCTGACAGATCTTCCTTCAGTTTTGTTGACAGAAAAGATTTGAAAACTATCTGACTTGAAAAATAATTTAATATCCATTCACTAAGGTCATTTACTTTCTCAGCATAGATACCGATTTTTGAATTGTCCCTTTTGGATTGCTGGGGAGGTTCTGCATTGATCCCATGCACCTTAGTAGTGGGTGAGAATTTTGACTTTCTTCTGTAAGGTGGGAGGACAGTTTTGAAAGGGGATGCTGTGTCCTCAGCTCCTTTCTCAAGCTGGGCAAGTTTAGAAAATAGAATATATAGCAATGGAAGATGTGGAAATGAATTCCCTTAAAACTGGTCTCCATAAACCCCAGGGGTAAGAGAACACCAGTTTGGAGCTTTAGGGGCTTTCTGGTCAAAGAGGGAATGGCCCAGGCGGTGATGTGGAGGATAGAATGAAGCACTGAGTATGAGTGGCGAAGGCGTACGGTGGGTGTAGAGATTTTCTGGGCGACGTGACCTAAGAAATGACTTTAGAACTGGCTAATCATTGGTTTTGGAGGGACTAAAAGTAGTTCCTGGTTGGTGAAAATAAATCATTAATGCGTTTTAAATGAAAAAGAAATGCATGCGTCTTGTAAAAAATGTGAAATAAAAGAGGCATAAAGTCAAAAGCAGAGCCTATGCCTTCCCCCACCAAACAAACAGGTAACCTAGTTAACAGGTGTGTCGTAGGATTCGAAGTTCGCCTCCAGCTCCGAGAGTGCGCCTACATCTCTTCCAACAAAATCCACTCACACTTGCAACGCCTGGCAAGGCTCTTCAATATGTGATTCCTTGACTGTCCTGTGCGTCTCTCGAGTGTAAACACCTCTGGGGCTCGTGATTCGGGGCTGCCAGAAAGAGAAGGAAGGTGCCGCCAGCGTCTGTTTCTTCCCGAAGTGAACTCCTACAACCTAGCCACCTTCTCCCCAGAGCTGTCGACTGGCTGTTGAAGGCCAATTTTTGTGCCTACGCAGGTCCTCAACACAGAACAAAACAAAAAAACAACAAAGGCCGGGCTAATAGCTATTTATAAACACTTACTGGACGCCCACTCTACGCCGAGCTCTCCCGCGCTCCTTGGATACTTTTTTGCAACGAGATGCCAATTTCCCCGGCGACCACTCCCTCAAACAGGCCTTCGCCTCCGCCCGCGCTGAGGCCCAGGCCCAGGTCCAGATTCAGAGCCGCCCGCCGGCTGGCGCTGCCCTGTAGGCGCCTGCGCAGAGCGACCCTCCCCGTCACTCGGAGCGGGAGGCGGGGGCAGCCGGGAGAAAGGAAAGCTGCGGGGGAAAAGGGCCAAACCCTGAAATTACCCGGATGTGGTCCCCGCGCGCGCATGCTCAGTGGCTTCTCGACAAGTTGGCAGCAACAACACGGCCCTGGTCGTCGTCGCCGCTGCGGTAACGGAGCGGTTTGGGTGGCGGAGCCTGCGTTCGCGCCTTCCCGCTCTCCTCGGGAGGCCC |
| **TCF4** | TTTCGCTAAATGTGTAAATCTAAAGGCAACCAAGTAAAAGACCTAAAAAAGGAAACTGTTAAGTCCATTTGTACATGGATTTAACAGTGAAGCATTTCATTCTTCATGTTTTCACTTGGTGTCTTGAAATAATCCCCTTTCTGTCTCCACTCTCCCTCTTAGACACTGCACCTGCCTTATTTATTTATGAAGTCTGAAGGTTAGTCCTACCTGCTGAGACTAATTAGCCCTGTGCTTTGTATCCTATGCAATCCTGTCTGATTAAATGTACACATTTGTTTTACTTCTTTTCAGGCTCCTTCTCACTCTACTTCTGATATTCTGATTAATATCAAAGTTGGGAGTTACAGTGATTCTCCTCTGCCTCCCCCCCAACCCATTTTTATTTTGCCTGCATCTTATTCTTTAGTGTGCAAATGTGGTTGTGATTTTTTTCCAG**TGCAGGGGAATTCGTTG**GC**CTT**GTCA**ATCTCGGTATCATTA**  **Mut: ATCTTGCTA**  **TT**TTTAGTAGTCTTTCTGAGACTTTAGCCTATAAAACAGTTTTGCTGAAACCCCAAACTCTCTAAATATTACATGGCAAATTTGCTTTCTTTTGGAAACCTCGGTGCTGATGGGCCAGCTGAAGTGCAGGTGAGCTTTTTCAGGGAAGGGTGGGACAACAGTACATCCTGGACTCTAGACTAATACTACTCCTAAGAGGCAATTTGCTAAATCCTACTGATTTATTTTTTTCTTTTTTCTCTTTTTCATGTTGTAGTGTCCAGTCTACTTCTTACTGGGTATTTGCAAAAATGAATTTTCTTGCTCTATCACCCAGGAACATTGTGTTTTGGGAGCTGGGTCTTTCCTCTTTAACATAATTCCATTCCCCATTTTGTAGCAAATCAGTCTGCTCCTCCTCTAATACCTAAAAGTCAACAAATACCTGCCTCAAGCCCCAGGTTTCCCATCTTCTCCCGAAGGTCTTCTAGCATCGGTTTGGTTAGCAATTTCCTGCCAGAGCCACCTACATCTTGATTATTTTGGTCAGTCTTTCGGGGATTTCATTTCAGACCCCCTTCGAGAAACACTGTAGCCCAACTGATCCCAGGGGTTGGTGAACTTTCAGTTTTATTTTCCCTGACGGTGTTAACGCCCAAACTTTTCACCTCCTAAGTCAAAAAGGACTTTGACTCTAGCTCATTTCCTACTACCTCCAACGTCAGTTAAAAAAAAAAAAAAAAAAGCAAAACACCACTCTACATTTAAAAGAATCATATATTTTTCCTTAAGGATACTTTTAATGTTTCTGACATTTAGCATTTGTTTTTTGCATAAAAAGTCGTTTTGGCATATCCATCCTAGTGGGACTTAACATTTCATGAAATGATTCACTACTAATAAACAAAAAAGGGAGGAGGGAGGCCTCATGGGTTAATAGTTTCTTTCATTGTAGATGACCAGGAACTTTGACCAGCCCCTTCACTTCCCCAAGCTCTCTGAAAGTGGAGGGTTGATCTTTCCTTTTGACCACTTTTGTCGCATCCCGTCTAAGGGTGGCTGATTTCACTGCTGAATTAACCACCAAGCACCCCCCCACCCCCTCCCCCAGCCACCACTTTCTCAAATACTACCCTTCCTTTTCCCCCTCCCTTAAGACTTATTTCTAATATTTCTAAAGTGCACTGTTTTGGGCCTCTCCCCATCCCCGCCCCCCAAGTGGGCTTTCCTTCCGCCTTCCTCGGCTCGGATTCCTGACTTGGTCGCCACCCCCTTCTCCTCCTCTCCCACCCCGCATTGTCTTTCTGAAACCGCCCCCTCCCGGAGCAAGTCCCTGCACCCTCGCCCAGAATCCCGGGCTCGCACACACTCCGCGCAGGCCGCTCCCCCTGCACACTCCTCCCTCCGTCTCCCCCCGGCTTCCCCGCCCCTCTCTTCCTCCTTCTTTCCCTCCTCCCTCTCCCGGCGCCCGAAAGGATCATTGTTAGCCGCCCCCGCCCCGCCCACCCCGGCTGTTTATTTATGCACACGTCACTGGGCCGGCCCCGCCCTCCGGCATCTCATTAAGGCAGTGTGTTCCTCTCGCCCTGTCAATAATCTCCG |
| **WNT11** | GTTTACTGACCTGAGCTCCAGGCTGGGGGCTAGGGAGGAGGAAAGTCCTGTCCCCGGCCCCGAGGCACATCTGCAACCGCCCGGAACATCTGGCTGAGCTCGCGCACGAGCTCCACGCCTCCCACCCCCGGGGCTGAAGTGCCTCGGTGTCCCTCAAGGCCCTGGGCGTGCAGGGTCCGGCTCCAGGGGGTTAGCAGGAGGGATCTCGAGTGCTCCCACCCACCCTTCCCACTTCCAAA**ACAACCCGTCTCCCGGGTGACCCGGCGCCGCGTGCGCAGCCAA**GCAGACGTCTCTCGAAAG  **Mut(ARE1): TAGCCCGAT**  CCTCACGGCTGGGGTCAAAGCCGGGTGGCAGGCCCGGCCTCGCCCCGCCCCGCAGGTAACCTTGAAGCCGTCG**CTCGGCGTCCCTGGTTGGCGGGGTCAGCTCCGTGAGCTGCGTG**GCGG  **Mut(ARE4): ATGGGGCTA**  GAGGCTTCGGGACCCGCCCGTGCCGACCTTGCGCGCCGCGCGGACAATCGGCCCCGGTGCACGGTGCCCGTGGGGCGTCGGGGTAGGGGGGACCCCGCAAGTCCTCGCCGGGCCGGCGTGGCCTGGTCCCAGCGGCCGGCTTTCCAGAGTCCTCCACAGACCGGGCCTATGACCATCTGTTGGCGGCGCTGGGCTGCGGCGCTGCGCTCGTGGTGTTCTCCCTGTGCACAGCCCCAGGCGGCCGCCTCCCGGGCCCGCTGCAGGCAGGGTAGCTCGGACCAACTTCGAGGAGGTGACCGCTCGCTCTCCAAAGGTGCTACGCCTGGCGCGGCCCACTACGCGCCCGGCCCCAAGCAAGGGGCCGGGCAGGTCAGGGCGGCCCACGGCAAGTGTCCTATAGAACGACCAGGACACAGGGGGCACTCCCTCGACACGTCGTCCCATCCCCAGACGGAGAAGCGGGGGCCCAGGAAGAGGACTTGACCCCAGCCCGCGACGCAGGAGGCAGCCGCGATTCCCCCCTCCTCGAGCCCCGGCTTAGCGCCCCCTGGCGGGCTGGGGGCTTCAGGAGG**CGCGTGCACCCTGACCCGCGAGCTCAGG**  **Mut(ARE5): ATGAGCCTA**  **CGGAAGGTGACCCCG**TGGCCTGCGCGGCTGTCCCGGGCGCTCGTACAAGGGACAGGAACAGTCCGTCAGGACCCAGAGGCTCGGGCCAGGCGCTGCCTCCCCGCGCGACGAGCTTGGGCGGCGGAGGGAGAGCCTTGGGACAGCCGGGGCTGCACCGAAAGAGCTGGGCGGGGCTGGAGGCCTGGGTCCCCGTCCGCTGTGCGACCTAGGGGGCTCCTCCTCTCCCTGGGAGACCAGGCTGG**ACACAGATCCCCCGCTGTGAGTCCGCGCGCCTCCGTCCTCTTG**CAGCCCCCGCCCCCT  **Mut(ARE2): TAGGTCCAT**  CCCCACCCAGGAATGCCGGGTGCCTTCGGGGGGCGGCGCACTTTGGGAGAGGGGAAGACGGTGACTGGAGTTTTGATTCCCCCCTCCATATCCAGCGGATATTTTTCTCGTTCTGAGAAGTGCAAGAAACTTGCAGGACCAGGAAGACAAAGGATTTGGATACAGGCTGGGTGGGAGGACGGGACTGGGTTACACCGAGAACCCCTCTGAGACCCCCAGCCTTGACGGCATGTTTGTCGCAGGCGCCCCTGTGCTGGGCGCTAGCTGCGGGGCTCTGCGGAGGAAAAAAACTGTACGATCCAGGCTTCTCCTCGCCTTCCTGTGGGTTTTGCCGACAGGCTCCAGAAAACAGTGGGGCTTCCCATCTCTGCGTGCTGACTACCCACGCGCAGGAACGCGCCACGCAGGTGGCGTCGCGGTGCAAATAGCAGAGGGGCGCTGAGCCCCGCAGGCTGCAGGGAGACGGGGTCCTCGGGGGACGCACGGCGAAGCCCCATTCCTCGTTCTTATGGAAGGGGACACCGAGGCTGGGGTGGCTGAGGGCTTGCCCAAGCTCACAGAGCCAGATAAGAACGTCCCTTTTCTGCTCCAGCGCCCCCACTACCGTGGGCGCCTCCTTTTCCAACTCCCCAAACCCTTAGAAAGGAATCCCTTTGTCTCTGCTCCTCCCGAGGGGAGAGGGGCTGGGGCTGTGTCTGACTCCGGGCGAACGCCAGGTGTTGGTAAATTTGGGAGAAGCTGGGAAGGGAGTTAATGAGGTGAGAATGAAGTGAAGGAGAAAAAAAGCGGAGATAGCTGCTCCGCGGGCTGCGGGCGGGCGGGGCAGCCTCTCCTCCGCGGCCCGAGGGCGCCGCCTGGAGGCCAGAGGCAACACCGCGCCAGCCCCGCGAGCTTCCCCGCTTCGCTAAGGCGGGGCTGCCCAGTCCCAGCCCGCATGCTTCCACTCCCACCCTCCAAAGTGGAGCGGCTAAAGACGGACTTGGAGACTCGGAATCCTCACTGTACAAAGAGCCCTACCATCCCGTTTCCCATCCTCCCCTTCCCACACCGAGAAAGCAGGGCCCCGAGCAAGCCCCATTTCCTGATTTCGCCGGACACCCGGCTTGCCGCTCCCTAGCTGAACCCCACCTCTGTGCCTCAG**TTTCCTCATCTGTGAGATGAGGCAGCGATAGTGCCTATCTCAC**GGGGTGCTGGGAGGAG  **Mut(ARE3): TAGGGCAAT**  CAGATGCGATCGTGTTAGGGAACTTTTTTTGTTTGTCTGTTTGTTTGAGACGGAGTTTCGCTCTTGTCGCCCAGGCTAGAGTGCAGTGGCGCAATCTCCGCTCGCTGCAACCTCCGCCTCCCGGGTTCAAGCGGTTCTCCTGCCTCAGCCTCCCGAGTAGCTGGGATTACAGGCGCCCGCCACCACGCCCGGCTAATTTTTTGTATTCGTAGTAGAGACGGGGTTTTGCCATGTTGGGCAGGCTGGTCTCGAGCTCCTGACCTCAGGTGACCCGCCCGCCTCGACCTCCCAAAGTGCTGGGATTACAGGCGTGAGCCACCCTGCCCGGCCAGGGAACGTTCTTTAT |
