# Supplementary material for: Nrf1 Is Endowed with a Dominant Tumor-Repressing Effect onto the Wnt/β-Catenin-Dependent and Wnt/β-Catenin-Independent Signaling Networks in the Human Liver Cancer
Source: Oxid Med Cell Longev. 2020 Mar 23;2020:5138539. doi: 10.1155/2020/5138539 (PMC7125503; doi:10.1155/2020/5138539)
Supplement: Supplementary 2 — Table S1: the key resources used in this work. Table S2: the sequencing data of genes encoding proteasomal subunits. Table S3: the sequencing data of genes involved in Wnt/β-catenin signaling pathway. Table S4: the promoters, the enhancer ARE/AP1-binding sequences, and the corresponding mutation sequences of the representative genes of Wnt/β-catenin signaling components. Table S5: the sequencing data of genes involved in the interactive network of Nrf1 interactors, migration and invasion pathways, carcinoma related pathways, signal transduction pathways, and metabolism pathways. Table S6: the sequencing data of DEGs whose RPKM values are greater than 3 in at least one cell line (shNC- or shNrf1-HepG2). Table S7: the sequencing data of genes implicated in the focal adhesion and ECM-receptor interaction. Table S8: the sequencing data of genes responsible for the pathways involved in cancer. Table S9: the promoters, the enhancer ARE-binding sequences, and the corresponding mutation sequences of PTEN, p53, CDH1, VAV1, PDGFB, and MMP9. [file 5138539.f2.zip › Table S9_ARE binding sequences in Figure S9B.docx]

| **Gene** | **Wild-type and mutant sequences of related gene promoters** |
| --- | --- |
| **PTEN** | GGCTGCAGCTTCCTACCGTTCCGTACTTTCCACTCAACCCGGTAACCCCAAACGTGCACGGTCCGGCCGGGGCGCGCGGAGCCTGGCCCCGGGCGATCCATCCTGCCGGGTTTTCACGGCGGCCAAGGGGGGGCGGGGCTAGGTGGTCTCTGAGAACCGAGCTTGACTCCGACGCCGCGAACCGACCTGGAGCCCGAGGGGAAAGATGCTCGACTCTCTTGGGGGCACCGGAGCGGGCGCAGGAGAGGCCTGCGGGGTGCGTCCCACTCACAGGGATCCTCTTTCAGTTCATTTAGATAGGTGCCCTTTGGGCCCTTGAAATTCAACGGCTATGTGTTCACGTTCAGCACGCTCGGCTGAGAGCTTTCATTTTTAGGGCAAACGAGCCGAGTTACCGGGGAAGCGAGAGGTGGGGCGCTGCAAGGGAGCCGGATGAGGTGATACACGCTGGCGACACAATAGCAGGTTGCTCTTTGTGCTAAGACTGACACCATGAGGACACAGATTTGGGGGAAGGGGGAATCTCTAGGCAAAGGCTGTTACAGTCAAATCTCTGCGAACGATTGTGATCCGACAGCGGTGCAAAAGGAAAGAGCGAATGCAGTCCACGCCGCGGAAATCTAGGGGTAGAGGCAAGGGGGGAGGGTATTCCCCTTGCAGGGACCGTCCCTGCATT**TCCCTCTACACTGAGCAGCGTGGTCACCTGGTCCTTTTCACCT**G  **Mut(ARE1): TTGTGGTCA**  TGCACAGGTAA**CCTCAGACTCGAGTCAGTGACACTGCTCAACGCACCCATCTCA**GCTTTC  **Mut(ARE2): TGACACTTT**  ATCATCAGTCCTCCACCCCCGCCCCACAACAGCCTACCCTGCCTCCGGCTGGGTTTCTGGGCAGAGGCCGAGGCTTAGCTCGTTATCCTCGCCTCGCGTTGCTGCAAAAGCCGCAGCAAGTGCAGCTGCAGGCTGGCGGCTGGGAACCGGCCCGAGCAAGCCCCAGGCAGCTACACTGGGCATGCTCAGTAGAGCCTGCGGCTTGGGGACTCTGCGCTCGCACCCAGAGCTACCGCTCTGCCCCCTCCTACCGCCCCCTGCCCTGCCCTGCCCTCCCCTCGCCCGGCGCGGTCCCGTCCGCCTCTCGCTCGCCTCCCGCCTCCCCTCGGTCTTCCGAGGCGCCCGGGCTCCCGGCGCGGCGGCGGAGGGGGCGGGCAGGCCGGCGGGCGGTGATGTGGCGGGACTCTTTATGCGCTGCGGCAGGATACGCGCTCGGCGCTGGGACGCGACTGCGCTCAGTTCTCTCCTCTCGGAAGCTGCAGC |
| **p53** | GGAGCCGCAGTCAGATCCTAGCGTCGAGCCCCCTCTGAGTCAGGAAACATTTTCAGACCTATGGAAACTGTGAGTGGATCCATTGGAAGGGCAGGCCCACCACCCCCACCCCAACCCCAGCCCCCTAGCAGAGACCTGTGGGAAGCGAAAATTCCATGGGACTGACTTTCTGCTCTTGTCTTTCAGACTTCCTGAAAACAACGTTCTGGTAAGGACAAGGGTTGGGCTGGGGACCTGGAGGGCTGGGGACCTGGAGGGCTGGGGGGCTGGGGGGCTGAGGACCTGGTCCTCTGACTGCTCTTTTCACCCATCTACAGTCCCCCTTGCCGTCCCAAGCAATGGATGATTTGATGCTGTCCCCGGACGATATTGAACAATGGTTCACTGAAGACCCAGGTCCAGATGAAGCTCCCAGAATGCCAGAGGCTGCTCCCCCCGTGGCCCCTGCACCAGCAGCTCCTACACCGGCGGCCCCTGCACCAGCCCCCTCCTGGCCCCTGTCATCTTCTGTCCCTTCCCAGAAAACCTACCAGGGCAGCTACGGTTTCCGTCTGGGCTTCTTGCATTCTGGGAC**AGCCAAGTCTGTGACTTGCACGGTCA**  **Mut(ARE1): TTACGGTCA**  **GTTGCCCTGAGGGGCTG**GCTTCCATGAGACTTCAATGCCTGGCCGTATCCCCCTGCATTTCTTTTGTTTGGAACTTTGGGATTCCTCTTCACCCTTTGGCTTCCTGTCAGTGTTTTTTTATAGTTTACCCACTTAATGTGTGATCTCTGACTCCTGTCCCAAAGTTGAATATTCCCCCCTTGAATTTGGGCTTTTATCCATCCCATCACACCCTCAGCATCTCTCCTGGGGATGCAGAACTTTTCTTTTTCTTCATCCACGTGTATTCCTTGGCTTTTGAAAATAAGCTCCTGACCAGGCTTGGTGGCTCACACCTGCAATCCCAGCACTCTCAAAGAGGCCAAGGCAGGCAGATCACCTGAGCCCAGGAGTTCAAGACCAGCCTGGGTAACATGATGAAACCTCGTCTCTACAAAAAAATACAAAAAATTAGCCAGGCATGGTGGTGCACACCTATAGTCCCAGCCACTTAGGAGGCTGAGGTGGGAAGATCACTTGAGGCCAGGAGATGGAGGCTGCAGTGAGCTGTGATCACACCACTGTGCTCCAGCCTGAGTGACAGAGCAAGACCCTATCTCAAAAAAAAAAAAAAAAAAGAAAAGCTCCTGAGGTGTAGACGCCAACTCTCTCTAGCTCGCTAGTGGGTTGCAGGAGGTGC |
| **CDH1** | AGACATTTCTGATCATTATTCCCATTAGGAGGGTGGAGAAACTGAGGCTTTGGGAGGTGGTCCTG**ACCTAGGGAATCAATTTGCTGACTCACTAACCCATGAAGCTCT**ACAGTTAAAAAA  **Mut(ARE1): TTTGACTCA**  GACTAGATTAAAAAATGAGAACTCAGTAAAGGGGCTGAGGCAGGAGGATCGCCTGAGTTCAGAAATTTGAGATCAGCCTCGGCAACATAGTGAGATCCCCTCTCTAGAAAAATTTTTTAAAAAATTAGGCCGCTCGAGGCAGAGTGCAGTGGCTCACGCCTGTAATCCAACACTTCAGGAGGCTGAAGAGGGTGGATCACCTGAGGTCAGGAGTTCCAGACCAGCCTGGCCAACATGGTGAAACCCCGTCTGTACTAAAAATACAAAATTAGCCGGTGTGGTGGCACACGCCTGTAGTCCCAGCTACTCAATAGGCTGAGACAGGAGAGTCTCTTGAACCCGGCAGGCGGAGGTTGCAGTGAGCCGAGATCGTGCCACTGCACTCCAGCCTGGGCAAGACAGAGCGAGACTCCGTCTCAAAAAATACAAACAAAACAAACAAACAAAAAATTAGGCTGCTAGCTCAGTGGCTCATGGCTCACACCTGAAATCCTAGCACTTTGGGAGGCCAAGGCAGGAGGATCGCTTCAGCCCAGGAGTTCGAGACCAGGCTGGGCAATACAGGGAGACACAGCGCCCCCACTGCCCCTGTCCGCCCCGACTTGTCTCTCTACAAAAAGGCAAAAGAAAAAAAAATTAGCCTGGCGTGGTGGTGTGCACCTGTACTCCCAGCTACTAGAGAGGCTGGGGCCAGAGGACCGCTTGAGCCCAGGAGTTCGAGGCTGCAGTGAGCTGTGATCGCACCACTGCACTCCAGCTTGGGTGAAAGAGTGAGACCCCATCTCCAAAACGAACAAACAAAAAATCCCAAAAAACAAAAGAACTCAGCCAAGTGTAAAAGCCCTTTCTGATCCCAGGTCTTAGTGAGCCACCGGCGGGGCTGGGATTCGAACCCAGTGGAATCAGAACCGTGCAGGTCCCATAACCCACCTAGACCCTAGCAACTCCAGGCTAGAGGGTCACCGCGTCTATGCGAGGCCGGGTGGGCGGGCCGTCAGCTCCGCCCTGGGGAGGGGTCCGCGCTGCTGATTGGCTGTGGCCGGCAGGTGAACCCTCAGCCAATCAGCGGTACGGGGGGCGGTGCCTCCGGGGCTCACCTGGCTGCAGCCACGCACCCCCTCTCAGTGGCGTCGGAACTGCAAAGCACCTGTGAGCTTGCGGAAGTCAGTTCAGACTCCAGCCCGCTCCAGCCCGGCCCGACCCGACCGCACCCGGCGCCTGCCCTCGCTCGGCGTCCCCGGCCAGCC |
| **VAV1** | CGTGGTGATGCATGCCCGTAATCTCAGCTACTTGGGAAGCTGAGACAGGAGAATCGCTTGAACCCAGGAGGCAGAGGTTGTGGTGAGCTGATCACACCACTGCACTCTAGCCTGGGCAACGAGAGTGAAACTCCGTCTCAAAAGAAAAAAAAAGAAGAAAGAAGCCTGGCGCGGTGGCTCACGCCTGTAATCCCAGCACTTTGGGAGGCTGAGGCGGGCGGATCATGAGATCGGGAGATCGAGACCACGATGAAACCCTGTCTCTACTAAAAATACAAAAAATTAGCCAGACGCGGTGGCGGACGCCTGTAGTCCCAGCTACTCAGGAGGCTGAGGCAGGAGAATGGCGTGAACCCGGGAAG**CGGAGCTTGCAGTGAACTGAGATTGCGCCACCGCACTCCAGCC**TGGGTGACAGAGTGA  **Mut(ARE1): TGAGATTTT**  GACTCCGTCTCAAAAAAAAAAAAAGAAGAAAGAAACAAAGAGAGAAAGAAAGGAAAGAAAGAAAGGAAGGAAGGAAGGAAGGAAGGAAGGAAGGAAGAAAGGAAGGGAGGGAAAGAGGGAGAGAAAGGAAGGAAGGAAAAAAATAACTTAAAAAATCAGATTTGTTGGACAAAGATCAGGGCTTAACCTAGGGAGGTGGGTAGAGCTAATGGAATGCGGAAAAGGCTGTGATTTGAAATGAGGGGATTTAGGAAGACCTCATGAGAAGGTAGCATTTGAGCAAAGACATGTAGGGGTGAGGGAGCTAGCCATGAAGTTGCTTAAGGTGGAGGACACAGCCCGTGCAAAGGCCCTGGGGCAGGGCCGTATGTTCCTGGCATGTTGGAGGAAGAGCGAAGAGGCCCGTGTGGCTGGAGCACAGTGAAGAGGGGGAGAGAGGGAGTGGGGAGGGCAGGGAGGGAACTGGGCAATTCAAGCAGGGTTTTGTGGGCCTTGGGGAGGACTTGGGCTTGTCCCTGGAGGAAAGTGGGAGCCATAGAGAGTTGTGGGCAGAAGAAGGGTGTGCCCTGACTCAGATGCTCACAGGCAACCTCTGGTGGTGGCTGCAGGGAGGACAGACTGTGGGGTACGGGGGCTGGAGTCA**GAAGACCAGCTGAGTGATGACGGGGCTGGACCAGACAGAGGAG**GGGGTGAGAAGTGGGTGAATTCTGGGTATATTTC  **TGACGGGTT :Mut(ARE3)**  AGAGTGTCACTGCCGCCGTCTGCATATGGAGGAAGCTCACCCATCTCATAGTCTAGCTGGCCTGACTCCCCCAGCCCCCCAACTCCCCATGCCCAGGCCTGTGTCGAGTGGGCGGAAGAAAGAGATGTCAGATTCTGCATGGAAGGCGTGGGGTGGGGCTGGGCTGCAGGTGCTCCCCCAGCTCCCCCCCGCCCCATGGCTCCTCCTCCTCCACCCCCTCTCAGGGCGACAGTTACAGGCAAAGAAGAGGAAGTGGTAGCACTAGCTGTCGCTCCACAGGCG |
| **PDGFB** | ACCCACGCACGTACACAGGCACGCACGGGCCCCCGTGCACCCAGCGCCTGGTGCTCGCCCCCGCGCAACAGGTGGGCCCTCCGTGGGCCCTGGCACCTCACCACCTCTGTAGCGGCCCCATTTCCTTCCTGGCGTCCTGTGAGGGAGGGAGAACCTCCCATCAGCACCACAG**CACCTACTTTTTTTTTTGCCTCGTCAGCCCGACGCCCCTCAAA**CCTTACCCATCTGTGACTCCTTTTT  **TTCTCGTCA :Mut(ARE1)**  TTCAACCACCTCCGCGTGGTGGAAAATGGTGGTGATGTGACTCTGAGGGGCACTGAGCTGTCCAGAGCGATTCCCCCTTCACATAAGCCTTCATTTGAACCTGCAAGACTGGAGGGACCTGGCGTGTGCAACCGCGGAGGGGGCTCCCACCCCTGGCTGTTGCATTCTCTTGGCTGATCCCAGCGTGCCCCGGGGAGGCCGCTGACAGCTGGATGTTTCCCCAGCCTCCCCTTACCATTTCCAGCTTCGTCCAGCACCTCCTCCTTCTTTCCCACAGCTCCACGGGCTCGTGTATCTGGGGTGGAGGCTGTGGCACAGAAACTGCCTTTCTCCTCACTTTAGTCACAGCATTCTTGAACACATGGCCACAGGCGCGATGTATGTGGCACTTTGCAGTTTATGAAGCACTTTGCTGCTAAGCCTGAGTGAGCCTCAGGCTGGCCCTGGGGGAGGGGACCTGCATGGGGATGGAACCACGCAGGGGTCAGTCCAGGAAGGAGCTGTAATGGCCAGTGCTGGGAGAGTCAGGGCAGGCCTGCTGGTGGAGGTGGCCTTGGAGCTGTCCACGTCCTGGTCGTGCTCGGACTAATCTTTCAGCAGACGGCAGGCAGCCGTGAGGCAGGGCTGGGTGGAGGGCCTGCCGAGGCCTCTGAGGTGCCATCTCCACCAGCTGAGCTGGCTTCCAGGAGGGCGAGTCCCACTGTCACGTGACGCGTCTGGCCTCAGCACACTTCTTCCGGGAAAGAGTGAAGGGCCCCACTGCCCTTTGCCATCCAGCTTCCTCTGGCTTTGCTAATGGCCCTAGGGGGCAGGAGACCAACTGCTGGAATCCCAGAGCCCTGGAGGTGTGCAAGGGCAGGTCAAACAGAATTTGGAGGATCTGGTGCAAGAGCCAGGAAGAGAGAGAGAGAGAGAGTGTGTGTGTGTGTGTGTGTGCGCATCTGAGAGAGAGAGAGAGAGAGACTGACTGAGCAGGAATGGTGAG |
| **MMP9** | GAAAGGGCTCCTATAGATTATTTTCCCCCATATCCTGCCCCAATTTGCAGTTGAAGAATCCTAAGCTGACAAAGGGGAAGGCATTTACTCCAGGTTACACTGCAGCTTAGAGCCCAATAACCTGGTTTGGTGATTCCAAGTTAGAATCATGGTCTTTTGGCAGGGTCTCGCTCTGTTGCCCAGGCTGGAGTGCAGTGACATAATCATGGCTCACTGTATCCTTGACCTTCTTTCTGGGCTCAAGCAATCCTCCCACCTCGGCCTCCCAAAGTGCTAAGATTACAGGAATGAGCCACCATACCTGGCCCTGAATCTTGGGTCTTGGCCTTAGTAATTAAAACCAATCACCACCATCCGTTGCGGACTTACAACCTACAGTGTTCTAAACATTTTATATGTTTGATCTCATTTAATCCTCACATCAATTTAGGGACAAAGAGCCCCCCACCCCCCGTTTTTTTTTTTACAGCTGAGGAAACACTTCAAAGTGGTAAGACATTTGCCCGAGGTCCTGAAGGAAGAGAGTAAAGCCATGTCTGCTGTTTTCTAGAGGCTGCTACTGTCCCCTTTACTGCCCTGAAGATTCAGCCTGCGGAAGACAGGGGGTTGCCCCAGTGGAATTCCCCAGCCTTGCCTAGCAGAGCCCATTCCTTCCGCCCCCAGATGAAGCAGGGAGAGGAAGCTGAGTCAAAGAAGGCTGTCAGGGAGGGAAAAAGAGGACAGAGCCTGGAGTGTGGGGAGGGGTTTGGGGAGGATATCTGACCTGGGAGGGGGTGTTGCAAAAGGCCAAGGATGGGCCAGGGGGATCATTAGTTTCAGAAAGAAGTCTCAGGGAGTCTTCCATCACTTTCCCTTGGCTGACCACTGGAGGCTTTCAGACCAAGGGATGGGGGATCCCTCCAGCTTCATCCCCCTCCCTCCCTTTCATACAGTTCCCACAAGCTCTGCAGTTTGCAAAACCCTACCCCTCCCCTGAGGGCCTGCGGTTTCCTGCGGGTCTGGGGTCTTGCCTGACTTGGCAGTGGAGACTGCGGGCAGTGGAGAGAGGAGGAGGTGGTGTAAGCCCTTTCTCATGCTGGTGCTGCCACACACACACACACACACACACACACACACACACACAC**ACACACACCCTGACCCCTGAGTCAGCACTTGCCTGTCAAGGAG**GGGTGGGGTCACAGGAGCGCCTCCTTAAAGCCC  **TGAGTCAGC :Mut(ARE2)**  CCACAACAGCAGCTGCAGTCAGACACCTCTGCCCTCACC |
